# A pharmacogenetic signature of high response to Copaxone in late-phase clinical-trial cohorts of multiple sclerosis

**DOI:** 10.1186/s13073-017-0436-y

**Published:** 2017-05-31

**Authors:** Colin J. Ross, Fadi Towfic, Jyoti Shankar, Daphna Laifenfeld, Mathis Thoma, Matthew Davis, Brian Weiner, Rebecca Kusko, Ben Zeskind, Volker Knappertz, Iris Grossman, Michael R. Hayden

**Affiliations:** 1 Faculty of Pharmaceutical Sciences, University of British Columbia, Vancouver, BC Canada; 2 BC Children’s Hospital, Department of Medical Genetics, University of British Columbia, Vancouver, BC Canada; 3 Immuneering Corporation, Cambridge, MA USA; 4 Teva Pharmaceutical Industries Ltd, Petach Tikva, Israel; 5 Teva Pharmaceutical Industries, Frazer, PA USA

**Keywords:** Pharmacogenetics, Copaxone, Glatiramer acetate, Treatment-response, Inter-individual variability, Multiple sclerosis, Multi-SNP signature, Multivariable Bayesian modeling

## Abstract

**Background:**

Copaxone is an efficacious and safe therapy that has demonstrated clinical benefit for over two decades in patients with relapsing forms of multiple sclerosis (MS). On an individual level, patients show variability in their response to Copaxone, with some achieving significantly higher response levels. The involvement of genes (e.g., HLA-DRB1*1501) with high inter-individual variability in Copaxone’s mechanism of action (MoA) suggests the potential contribution of genetics to treatment response. This study aimed to identify genetic variants associated with Copaxone response in patient cohorts from late-phase clinical trials.

**Methods:**

Single nucleotide polymorphisms (SNPs) associated with high and low levels of response to Copaxone were identified using genome-wide SNP data in a discovery cohort of 580 patients from two phase III clinical trials of Copaxone. Multivariable Bayesian modeling on the resulting SNPs in an expanded discovery cohort with 1171 patients identified a multi-SNP signature of Copaxone response. This signature was examined in 941 Copaxone-treated MS patients from seven independent late-phase trials of Copaxone and assessed for specificity to Copaxone in 310 Avonex-treated and 311 placebo-treated patients, also from late-phase trials.

**Results:**

A four-SNP signature consisting of *rs80191572* (in *UVRAG*), *rs28724893* (in *HLA-DQB2*), *rs1789084* (in *MBP*), and *rs139890339* (in *ZAK(CDCA7)*) was identified as significantly associated with Copaxone response. Copaxone-treated signature-positive patients had a greater reduction in annualized relapse rate (ARR) compared to signature-negative patients in both discovery and independent cohorts, an effect not observed in Avonex-treated patients. Additionally, signature-positive placebo-treated cohorts did not show a reduction in ARR, demonstrating the predictive as opposed to prognostic nature of the signature. A 10% subset of patients, delineated by the signature, showed marked improvements across multiple clinical parameters, including ARR, MRI measures, and higher proportion with no evidence of disease activity (NEDA).

**Conclusions:**

This study is the largest pharmacogenetic study in MS reported to date. Gene regions underlying the four-SNP signature have been linked with pathways associated with either Copaxone’s MoA or the pathophysiology of MS. The pronounced association of the four-SNP signature with clinical improvements in a ~10% subset of the MS patient population demonstrates the complex interplay of immune mechanisms and the individualized nature of response to Copaxone.

**Electronic supplementary material:**

The online version of this article (doi:10.1186/s13073-017-0436-y) contains supplementary material, which is available to authorized users.

## Background

Multiple sclerosis is a chronic progressive disorder of the central nervous system, with a complex pathogenesis and polygenic inheritance [1]. Recent genetic studies of multiple sclerosis have found several hundred commonly occurring non-coding polymorphic loci to be associated with susceptibility to the disease [2–6]. Genetic polymorphisms within the human leukocyte antigen (*HLA*) region account for approximately 10% of the genetic risk of multiple sclerosis, with the HLA-DRB1*15:01 allele associated with a disproportionately elevated risk of developing the disease [7, 8]. Genetic influences on disease progression and severity remain an active area of research [9]. Fourteen disease-modifying therapies (DMTs) are currently approved for management of multiple sclerosis in the USA [10], benefiting patients by reducing relapses, delaying disability progression and reducing central nervous system lesions. These therapies vary in their mechanism of action (MoA), administration routes, and side effect profiles, with patients demonstrating substantial variability in their responses to each drug [11]. This variability, together with the plethora of treatment options, underscores the need for predictive markers of response to optimize treatment selection for individual patients of multiple sclerosis [11].

Copaxone® (glatiramer acetate) is a complex mixture of numerous polypeptides, each giving rise to several antigens that beneficially modulate the immune system through mechanisms that have not yet been fully unraveled [12–14]. It has consistently demonstrated an annualized relapse rate (ARR) reduction of ~30% in Copaxone-treated patients compared to those treated with placebo in clinical trials. It continues to be an efficacious treatment for multiple sclerosis with a favorable safety profile demonstrated over 20 years of clinical use and over two million patient-years of exposure [15]. Studies have shown that a large proportion of Copaxone-treated patients (38 to 56%) demonstrate high response, based on varying response definitions [11]. The involvement of genes with high inter-individual variability in Copaxone’s MoA [16–18] together with past research findings [19] suggest that genetic determinants may contribute to variability in Copaxone-response [11, 20].

To date, pharmacogenetic studies of Copaxone, ranging in size from tens to a few hundreds of patients (Additional file 1), have been based on candidate-genes presumed to be associated with its MoA, e.g., production and activation of Copaxone-specific anti-inflammatory and regulatory T-cells [16–19, 21]. The presence of variants in the *HLA* class II genes has been observed to be positively associated with Copaxone response. Examples of such variants include the DRB1*1501 allele [16, 17] or the homozygous presentation of a haplotype derived from the *DR15* and *DQ6* alleles along with absence of the *DR17* and *DQ2* alleles [18]. In contrast, allelic combinations of HLA DRB1*15, TGFB1*T, CCR5*d, and IFNAR1*G have been associated with non-response [22]. Alleles in other non-*HLA* genes such as T-cell receptor beta (*TRB*), Cathepsin S (*CTS*), Myelin basic protein (*MBP*), Cluster of differentiation 86 (*CD86*), Interleukin-1 receptor 1 (*IL1R1*), and *IL12RB2* have also been linked to Copaxone response with varying strengths of association [19]. While these candidate-gene studies have increased our understanding of the pharmacogenetics of Copaxone response and highlighted the potential importance of immune-response genes in Copaxone therapy, these findings have not been replicated. Furthermore, a comprehensive and simultaneous assessment of the contribution of multiple gene variants to Copaxone response has not been performed.

The current study is the largest pharmacogenetic study in multiple sclerosis reported thus far (Additional file 1), identifying and independently assessing a genetic signature associated with Copaxone response in patient cohorts from a series of multinational late-phase clinical trials. The study design included signature identification using an initial exploratory association analysis of genome-wide SNP data informed by published research, Bayesian predictive modeling, independent assessment of the signature for performance and specificity, and finally, clinical characterization of patient subsets delineated by the signature.

## Methods

### Study design

**Fig. 1:**
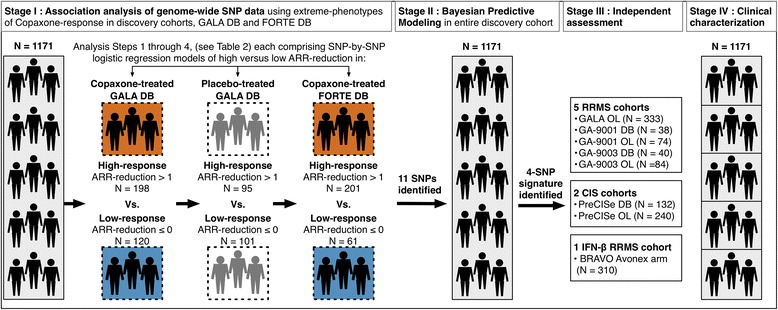
Study design. The four stages of the study design are shown in sequence along with the sample sizes of each of the trial cohorts utilized in the study. *DB* double-blind, *OL* open-label; *RRMS* relapsing-remitting multiple sclerosis

A four-stage study design was employed to identify a multi-SNP signature for Copaxone response (Fig. 1). In stage I, genome-wide SNP data were used to identify SNP-by-SNP associations with extreme phenotypes of Copaxone response in 318 Copaxone-treated patients from the GALA study [23]. Identified SNPs were examined in 196 placebo-treated GALA patients to filter out prognostic markers and then screened for association with extreme phenotypes of Copaxone response in 262 Copaxone-treated patients from the FORTE study [24]. In stage II, multivariable Bayesian modeling was employed to identify a multi-SNP signature correlated with response from among the SNPs selected in stage I. A combined cohort of 1171 patients from the GALA and FORTE studies was employed for modeling. The signature was tested in 311 placebo-treated patients from the GALA study to confirm its non-prognostic nature. In stage III, the identified multi-SNP signature was assessed in seven independent late-phase trial cohorts, as well as in a cohort treated with Avonex (IFN-β) to test for specificity to Copaxone. In stage IV, patient subsets defined by the multi-SNP signature were characterized to identify trends in clinical measures indicative of disease progression.

### Study populations

#### Discovery cohorts

The discovery cohorts included patients with relapsing-remitting multiple sclerosis (RRMS) from two large phase III double-blind (DB) clinical trials of Copaxone (Fig. 1, Table 1).

**Table 1:** Demographics and baseline clinical characteristics of the study population

	Discovery		Independent assessment							Specificity assessment
	GALA DB	FORTE DB	GALA OL	GA-9001 DB	GA-9001 OL	GA-9003 DB	GA-9003 OL	PreCISe DB	PreCISe OL	BRAVO Avonex arm
Type of multiple sclerosis	RRMS	RRMS	RRMS	RRMS	RRMS	RRMS	RRMS	CIS	CIS	RRMS
Phase of trial	Phase III	Phase III	Phase IV	Phase III	Phase IV	Phase III	Phase IV	Phase III	Phase IV	Phase III
Nationality	Multinational	Multinational	Multinational	US	US	Multinational	Multinational	Multinational	Multinational	Multinational
Number of patients	639	532	333	38	74	40	84	132	240	310
Duration of follow-up	1 year	1 year	~3 years^a^	~3 years	~20 years^a^	0.75 years	0.75 years	3 years	5 years	2 years
Age (mean ± SD)	37.59 ± 9.33	36.19 ± 8.76	38.5 ± 9.25	35.89 ± 5.40	36.35 ± 5.92	33.33 ± 7.75	33.46 ± 7.65	31.71 ± 7.25	32.08 ± 7.15	38.17 ± 9.28
Sex (percentage female)	69.01%	72.37%	67.57%	68.42%	71.62%	70.00%	76.19%	63.64%	63.75%	68.06%
Caucasian (%)	97.81%	100.00%	99.70%	92.11%	90.54%	97.50%	97.62%	96.21%	97.50%	98.71%
Baseline EDSS	2.79 ± 1.22	2.14 ± 1.11	2.86 ± 1.32	2.79 ± 1.34	2.66 ± 1.62	2.14 ± 0.99	2.31 ± 1.32	0.99 ± 0.82	1.27 ± 1.08	2.62 ± 1.15
Baseline ARR	0.93 ± 0.45	0.98 ± 0.44	0.88 ± 0.50	1.49 ± 0.56	1.05 ± 0.78	1.21 ± 0.78	1.25 ± 0.78	NA	NA	0.94 ± 0.45

Copaxone doses: GALA DB, 40 mg/mL thrice-a-week; Forte DB, both 20 mg/mL and 40 mg/mL a day arms were included; GALA OL, 40 mg/mL a day; GA-9001, GA-9003, and PreCISe, 20 mg/mL a day.

^a^The follow-up is ongoing and the values represent the time-point at which the data were summarized for this study.

*Avonex* interferon β-1a, *Baseline ARR* individual ARR for two years prior to study, *CIS* clinically isolated syndrome, *EDSS* Kurtzke expanded disability status scale, *OL* open-label, *RRMS* relapsing-remitting multiple sclerosis.

Only patients who gave their informed consent to being genotyped were included in the study. Genotyped patients were representative of the study population in the parent trial

##### GALA DB

[23]: The *Glatiramer Acetate Low-frequency Administration* study (ClinicalTrials.gov: NCT01067521) compared a three-times-a-week regimen of 40 mg/mL Copaxone with placebo. The duration of the DB phase was 12 months. It was conducted at 142 sites in 17 countries, including Bulgaria, Croatia, Czech Republic, Estonia, Georgia, Germany, Hungary, Israel, Italy, Lithuania, Poland, Romania, Russia, South Africa, Ukraine, United Kingdom and United States. In stage I, 318 Copaxone-treated patients from the DB phase of the GALA study were analyzed (Fig. 1); 196 placebo-treated patients from the DB phase of the GALA study were assessed to filter out prognostic markers. Subsequently, 639 total patients from the study were genotyped in stage II. Only the Copaxone arms were used in multi-SNP modeling.

##### FORTE DB

The *FORTy mg Efficacy of glatiramer acetate* study (ClinicalTrials.gov: NCT00337779) compared once-daily doses of 20 mg/mL to 40 mg/mL Copaxone [24]. The duration of the DB phase was 12 months. It was conducted at 136 sites in 20 countries including Argentina, Belgium, Canada, Czech Republic, Estonia, Finland, France, Germany, Hungary, Israel, Italy, Latvia, Lithuania, Netherlands, Poland, Romania, Russia, Spain, the UK, and the US. The study concluded that the mean number of relapses were equivalent at both doses [24]. Both arms were thus pooled for analysis. In stage I, 262 Copaxone-treated patients from the study were analyzed (Fig. 1). Subsequently, a total of 532 patients from the study were genotyped in stage II.

#### Independent assessment cohorts

The independent assessment cohorts included patients from an additional independent set of RRMS clinical trials and one study in clinically isolated syndrome (CIS). Data from these cohorts were utilized to assess the multi-SNP signature in stage III.

##### GALA OL

The open-label (OL) phase of the GALA study (ClinicalTrials.gov: NCT01067521) comprised 311 patients who were on placebo treatment in the DB phase and were switched to three times-a-week Copaxone 40 mg/mL once the DB phase was completed (“delayed start cohort”) [25]. The OL phase of the study is ongoing at the same sites and countries as the GALA DB phase (see “GALA DB” section above). The placebo-response data from these patients, prior to the switch to active treatment, was used to filter out prognostic markers in stage I and also confirm the non-prognostic nature of the multi-SNP signature in stage III.

##### GA-9001

Thirty-eight Copaxone-treated patients from the placebo-controlled DB phase (ClinicalTrials.gov: NCT00004814) and 74 delayed-start Copaxone-treated patients from the OL phase (ClinicalTrials.gov: NCT00203021) were included [26]. The duration of the DB phase of the study was 35 months (initially 24 months, and later extended 11 additional months) [27]. It was conducted in 11 sites in the US. The OL phase is ongoing at the same sites as the DB phase.

##### GA-9003

For the GA-9003 cohort [28] eighty-four Copaxone-treated patients from the placebo-controlled DB phase of the *European-Canadian Copaxone trial* and 40 delayed-start Copaxone-treated patients from the OL phase [29] of the study were included. The duration of the DB and OL phases of the study was 9 months each, and both were conducted at 29 sites in seven countries including Belgium, Canada, France, Germany, Italy, Netherlands, and the UK.

##### PreCISe

The *early glatiramer acetate in patients Presenting with a Clinically Isolated Syndrome* study (ClinicalTrials.gov: NCT00666224) [30] demonstrated the efficacy of Copaxone in delaying the progression of unifocal CIS to clinically defined RRMS. The duration of the DB phase was 36 months and that of the OL phase 60 months. Both were conducted at 80 sites in 16 countries including Argentina, Australia, Austria, Denmark, Finland, France, Germany, Hungary, Italy, New Zealand, Norway, Romania, Spain, Sweden, the UK, and the US.

The study cohort comprised 132 Copaxone-treated patients from the placebo-controlled DB phase and 240 delayed-start Copaxone-treated patients from the OL phase [31].

##### BRAVO

The *Laquinimod DB placebo controlled study with a rater-Blinded Reference Arm of interferon β-1a (AVOnex®)* (ClinicalTrials.gov: NCT00605215) [32] compared the effect of Laquinimod with that of Avonex for RRMS patients. The duration of the DB phase was 24 months. It was conducted in 155 sites in 18 countries including Bulgaria, Croatia, Czech Republic, Estonia, Georgia, Germany, Israel, Italy, Lithuania, Macedonia, Poland, Romania, Russia, Slovakia, South Africa, Spain, Ukraine, and the US. The Copaxone specificity of the multi-SNP signature was evaluated using response data from 310 patients from the Avonex arm of the trial. Inclusion criteria and assessment frequency were similar to the other Copaxone studies.

Baseline demographics as well as clinical characteristics of the patient cohorts included in this study (Table 1) are representative of parent trial populations and are within one standard deviation for continuous measures and had similar percentages for categorical measures [23–32].

## Response definitions

### Extreme phenotypes of Copaxone response

For each patient, ARR-reduction was calculated as the difference between ARR during the study and ARR for the two years prior to the study. For patients with fewer than two years of recorded clinical history, time since the first symptom (in years) was used when calculating the pre-study ARR. To determine the extreme phenotypes of Copaxone response, the distribution of ARR reduction was examined in the discovery cohorts and cut-off thresholds were selected to define high and low response categories (Fig. 1). An ARR reduction >1 was defined as high and patients meeting this definition were classified as *high responders*. The *highest responders* were patients with high ARR reduction and, additionally, no new T2-weighted brain MRI lesions (T2 lesions). Patients with an ARR reduction of ≤0 (i.e., no ARR reduction or a worsening of ARR) were classified as *low responders*. The *lowest responders* were patients with low ARR reduction and, additionally, one or more new T2 lesions. Patients with an ARR reduction between 0 and 1 inclusive were considered intermediate responders and not genotyped in stage I.

### Relapse-free definition

Subsequent to identification of genetic variants correlated with extreme phenotypes of Copaxone response, predictive models were built on the most clinically useful response definition as indicated by treating physicians, i.e., being relapse-free. Only patients with at least one relapse at baseline or a baseline ARR ranging from 0.5 to 1.0, given one to two years of available clinical history, were included in the parent trials. Therefore, being relapse-free during the trial was assumed to be a treatment effect. A patient was considered relapse-free or a responder if he or she did not experience any relapses within one year of starting treatment. As a result, the relapse-free definition captured all patients with an ARR reduction between 0.5 and 1 inclusive as well as those classified as intermediate responders (see section above). Ninety-seven patients experiencing a relapse within the first 47 days after starting therapy were excluded because DMTs, and specifically Copaxone, do not reach full efficacy until after this period [33]. Therefore, relapses in the first 47 days were not considered as failures of drug response. Sensitivity analysis indicated that results were not affected by this exclusion.

### Genotyping and quality control

The Illumina OMNI-5M genome-wide array covering 4,301,331 SNPs was utilized for genotyping the patients with extreme phenotypes of Copaxone response in stage I. Genotypes were called with the Illumina Genome Studio software and their quality checked with evaluations for call rate, cluster separation, mean normalized intensity, proximity of heterozygote clusters to a homozygote cluster, heterozygous excess, false homozygosity, and reproducibility-related errors. SNPs with call rates of ≥95% were retained and those with <95% were either re-clustered or removed. Deviation of genotype distributions from Hardy–Weinberg equilibrium (HWE) was tested in placebo arms of the discovery cohorts. SNPs with a *p* value <1.0 × 10^−4^ for Fisher’s exact test for HWE were excluded. A total of 4,296,423 SNPs were retained after the quality control steps outlined above.

SNPs identified at the end of stage I were genotyped using TaqMan SNP genotyping assays in 1171 patients from GALA DB and FORTE DB cohorts, and in an additional 941 patients from GALA OL, GA-9001, GA-9003, PreCISe, and BRAVO cohorts. Cluster plots for these SNPs were visually inspected. A small number of samples (n = 16) underwent confirmatory Sanger sequencing for all SNPs. Within these samples, there was 100% concordance between the genotypes called by the OMNI-5M genome-wide array, the TaqMan assay, and Sanger sequencing, regardless of minor allele frequency.

## Statistical methods

### Association analysis

Stage I was an initial exploratory analysis to identify candidate SNPs for follow-up in later stages. For each SNP, a logistic regression model was built using response variables based on the extreme phenotypes of Copaxone response. Models were estimated using SVS software, version 8.3.0. A four-step analysis (Table 2) incorporated a priori evidence in the SNP selection process. Step 1 employed regression models to select SNPs from a set of 35 candidate variants supported by prior literature (Additional file 2). Subsequent steps expanded the set of SNPs tested in a non-overlapping manner, ending in a broad genome-wide analysis (Table 2). Since the purpose of this stage was hypothesis generation, lenient thresholds were adopted at each step to capture SNPs based on both strong biological plausibility and pre-existing literature evidence. Candidate variants and genes analyzed in steps 1 and 2 are listed in Additional file 2.

**Table 2:** Association analysis of genome-wide SNP data in patients with extreme-phenotypes of Copaxone-response

Analysis steps and inclusion thresholds	Selected SNPs		Copaxone-treated patients			
			GALA DB		FORTE DB	
	Gene	SNP rsID	Odds ratio	*P* value	Odds ratio	*P* value
Step 1. Replicated variants from 35 prioritized candidate variants. Inclusion threshold: *p* value <0.05 GALA, *p* value <0.05 FORTE	HLA-DRB1*1501	*rs3135391*	0.66	0.040	0.64	0.0499
Step 2. Priority list of 4012 variants in 30 genes. Inclusion threshold: *p* value <0.05 GALA, *p* value <0.05 FORTE	*HLA-DQB2/DOB*	*rs28724893*	0.53	0.00060	0.46	0.00037
	*HLA-DOB/TAP2*	*rs1894408*	1.72	0.0030	1.82	0.0093
	*MBP*	*rs1789084*	0.70	0.036	0.57	0.01
Step 3. Broad genome-wide analysis. Inclusion threshold: *p* value <0.01 GALA, *p* value <0.05 FORTE	*PTPRT*	*rs117602254*	0.21	0.0037	0.28	0.016
	*ALOX5AP*	*rs10162089*	1.56	0.0078	1.58	0.032
	*MAGI2*	*rs16886004*	2.15	0.0023	5.56	3.3E-05
	*ZAK(CDCA7)*	*rs139890339*	0.05	3.4E-05	0.14	0.011
	*SLC5A4(RFPL3)*	*rs73166319*	*	0.0060	*	0.015
Step 4. Secondary genome-wide screen in patients with highest Copaxone response (relapse-free with no new T2 lesions). Inclusion threshold: *p* value <0.01 GALA, *p* value <0.05 FORTE	*UVRAG*	*rs80191572*	0.20	0.0024	0.12	3.4E-05
	*SLC1A4*	*rs759458*	3.31	4.4E-05	1.86	0.049

The 35 prioritized candidate variants and the 30 genes analyzed in steps 1 and 2, respectively, are presented in Additional file 2. SNPs selected at each analysis step met the indicated threshold of significance in the SNP-by-SNP logistic regression models built separately in the GALA DB and the FORTE DB cohorts. These models estimated the odds ratios of high versus low response. The SNPs that were selected at each step were not associated with the extreme phenotype of response in patients treated with placebo. *Odds ratios were not informative since the rare allelic variant of *SLC5A4(RFPL3*) was only present in high responders of Copaxone treatment and not in low responders. *DB* double-blind phase, *MAF* minimum allelic frequency

### SNP encoding and inheritance models

The inheritance model for each SNP was determined using PLINK [34]. Each SNP was coded either as a continuous covariate with values 0, 1, and 2, denoting an additive inheritance model, or as a binary variable with two levels, 0 and 1, denoting a dominant inheritance model that specified whether or not a patient had two copies of the minor allele. The frequencies for each genotype of the four SNPs in the four-SNP model are shown in Additional file 3.

### Predictive modeling

In stage II, logistic regression models were built, both with and without disease-related baseline covariates, to determine which of the SNPs identified in stage I were most predictive of relapse-free status. Covariates consisted of the baseline Kurtzke Expanded Disability Status Scale (EDSS) [34], Log (number of relapses for past two years + 1), baseline T2 lesion volume, and gadolinium-enhancing T1-weighted MRI lesion (T1 lesion) status (0 for no lesion, 1 for at least one lesion at baseline). The logistic regression models were estimated using Bayesian model averaging (BMA) [35–39] with a spike-and-slab prior distribution [40], as implemented in the BoomSpikeSlab R package [41]. The sparsity-inducing spike-and-slab prior in this model embodied the expectation that not all identified SNPs from stage I were important for predicting relapse-free status. Model convergence diagnostics for BMA were inspected to determine that 50,000 iterations of the Markov chain Monte Carlo (MCMC) sampler were sufficient to explore the space of all possible SNP combinations and, hence, estimate the final BMA model [40–42]. For each SNP, BMA provided the posterior probability of inclusion in the model and the 95% Bayesian confidence interval (CI) of the posterior distribution of its regression coefficient. SNPs were deemed statistically significant if the 95% CI of their regression coefficients did not include zero, both with and without the inclusion of disease-related baseline covariates in the model. The strength of evidence for each SNP’s effect was assessed quantitatively by computing its posterior probability of inclusion in the model and qualitatively by examining the width of the 95% CI of its regression coefficient. Potential interactions were assessed between those SNPs whose main effects were significant. The final model was obtained by refitting the statistically significant SNPs without baseline covariates. The model-building process and the rationale behind the choice of the Bayesian framework are described in detail in Additional file 4.

### Classification performance

Stage III evaluated the classification performance of the multi-SNP signature resulting from stage II analyses in each of the independent cohorts using sensitivity, specificity, and the area under the receiver operating characteristic (ROC) curve (AUC). To classify patients as either relapse-free or relapsing, an optimal threshold on the predicted probabilities from the multi-SNP logistic regression model was determined. This threshold maximizes the sensitivity and specificity of the signature and corresponds to the point on the ROC curve closest to the top left corner (“top-left" threshold). Signature-positive patients were those who either met or exceeded the predicted probability that corresponded to the “top-left” threshold in the multi-SNP model.

### Clinical characterization

**Fig. 2:**
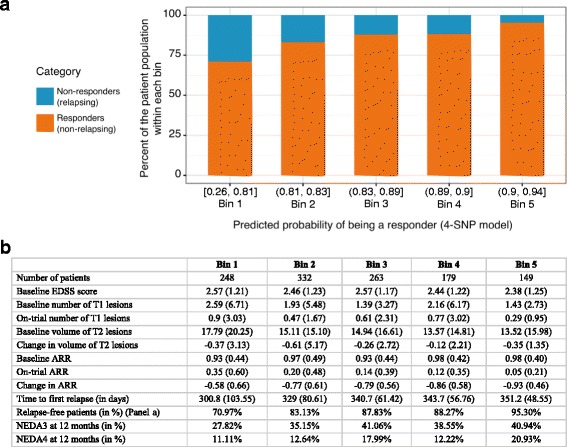
Clinical characterization of patients in the discovery cohort. **a** Proportion of relapsing and non-relapsing patients across bins in the discovery cohort. **b** Clinical characterization of patients within each bin in the discovery cohorts. Panels **a** and **b** show descriptive summaries of clinical characteristics relevant to disease progression across five patient bins. These bins were constructed using the logistic regression model which predicted the probability of being relapse-free conditional on the four SNPs. In **a**, each *tick* on the *x-axis* corresponds to a bin based on a quintile of the predicted probability from the logistic regression model and is labeled with the lowest and the highest predicted probability for the bin. As we move from left to right along the *x-axis*, the predicted probability of being relapse-free (or being a responder) increases. In each *bar*, the observed percentages of non-responders and responders are shown using two colors. For a good model, the predicted probabilities should be close to the observed percentages. The figure confirms that this is indeed the case for the logistic regression model. The *bars* in the graph in **a** and the columns of the table in **b** are lined up to show the one-to-one correspondence between the graph and the table. Panel **b** illustrates that the trends of several alternative clinical response definitions which were not used to construct the four-SNP model align well with the predicted probabilities from the four-SNP model. This suggests that the predictive value of the four-SNP genotype extends beyond the clinical response definitions used to build it. *T1 lesions* are gadolinium-enhancing T1-weighted lesions on MRI; *T2 lesions* are T2-weighted MRI lesions. *ARR* annualized relapse rate, *NEDA3* no evidence of disease activity (version 3), *NEDA4* no evidence of disease activity (version 4). Percentages of patients meeting the NEDA3 and NEDA4 definitions are shown. The discovery cohorts consisted of the patients from GALA DB and FORTE DB

In stage IV, patients in the discovery cohorts were divided into five similar-sized bins (Fig. 2) based on quintiles of the predicted probabilities from the multi-SNP model. The distribution of key clinical measures was assessed across these bins. For each bin, descriptive summaries, including the mean and standard deviation for continuous variables and the percentage of patients in the category of interest for categorical variables, were calculated for baseline measures (EDSS score, number of T1 lesions, T2 lesion volume, ARR) and for on-treatment measures (on-trial number of T1 lesions, change in volume of T2 lesions, on-trial ARR, change in ARR, time to first relapse (in days), percentage of patients who were classified as non-relapsing (responders) and percentage of patients meeting the two definitions of *no evidence of disease activity* (NEDA3 and NEDA4)) [43], computed at 12 months after initiation of treatment. NEDA3 consisted of three criteria: (a) no relapse; (b) no confirmed disease progression defined as a 1-point increase of EDSS from baseline for patients with baseline EDSS between 0 and 5, or a 0.5 increase for patients with baseline EDSS higher than 5, confirmed 3 months later; and (c) no T1-weighted gadolinium-enhancing lesions or new or enlarging T2 lesions measured by MRI during the study. NEDA4 additionally included brain volume loss of ≥0.4% as a criterion. Clinical characteristics of signature-positive and signature-negative patients were also compared.

## Results

### Eleven SNPs were associated with extreme phenotypes of Copaxone response

Eleven SNPs were associated with high versus low response to Copaxone based on the initial exploratory analysis of genome-wide SNP data from the patients in both the GALA DB and FORTE DB discovery cohorts (“Methods”, Table 2). These SNPs were not associated with response in the GALA DB placebo arm.

Briefly, in step 1 of the analysis, out of 35 candidate variants tested, two SNPs in complete linkage disequilibrium (LD) (*rs3135391*, *rs3135388*) tagging the HLA-DRB1*15:01 allele met the threshold for selection in both GALA DB and FORTE DB. *rs3135391* was selected for all subsequent analyses. In step 2, out of 4012 variants in 30 candidate genes, 36 variants were selected in both discovery cohorts. Of these variants, three SNPs not in LD with each other and located in the *HLA-DQB2/DOB*, *HLA-DOB/TAP2*, and *MBP* gene regions, respectively, were selected. In step 3, a broad genome-wide analysis identified five SNPs located in the *PTPRT*, *ALOX5AP*, *MAGI2*, *ZAK/CDCA7*, and *RFPL3/SLC5A4* gene regions, respectively. In step 4, a broad genome-wide analysis limited to the patients with the highest response (defined as relapse-free with no new T2 lesions) identified two SNPs in the *UVRAG* and *SLC1A4* gene regions, respectively.

### A four-SNP signature was associated with the binary relapse-free response definition

From among the 11 SNPs associated with extreme phenotypes of Copaxone response (stage I), Bayesian predictive modeling in 1171 patients comprising the broader discovery cohort identified a subset of four SNPs (Table 3) that distinguished relapse-free patients (responders) from relapsing patients (non-responders). Each of these four SNPs attained a >80% posterior probability of inclusion in the model with statistically significant effects (i.e., the 95% CI of the posterior distribution of their regression coefficients did not include zero). In contrast, the remaining seven SNPs which were not selected had posterior inclusion probabilities smaller than 60% and non-significant effects. Additional file 5 presents the regression coefficients and the posterior inclusion probabilities of the 11 SNPs. Interactions between the top four SNPs had posterior inclusion probabilities of <1% and were thus not included in the final model.

**Table 3:** The four-SNP model coefficients and odds ratios with 95% Bayesian confidence intervals

SNP rsID	Gene	Regression coefficient (95% CI)	Odds Ratio (95% CI)
*rs80191572*	*UVRAG*	−0.68 (−1.06, −0.29)	0.50 (0.35, 0.75)
*rs28724893*	*HLA-DQB2*	−0.52 (−0.75, −0.29)	0.59 (0.47, 0.75)
*rs1789084*	*MBP*	−0.61 (−0.98, −0.25)	0.54 (0.38, 0.78)
*rs139890339*	*ZAK(CDCA7)*	−1.46 (−2.31, −0.63)	0.23 (0.10, 0.53)

Coefficients of the four-SNP model were obtained by fitting a logistic regression model on data from the patients treated with Copaxone in the GALA DB and FORTE DB studies. The SNP from *MBP* was coded according to a dominant inheritance model. All the other SNPs were coded according to an additive inheritance model. The logistic regression model estimated the log odds of being relapse-free conditional on the four SNPs. A negative regression coefficient for a given SNP implies that the major allele (coded as the reference level in the logistic regression model) was associated with increased odds of being relapse-free while the minor allele was associated with increased odds of relapses

### Signature-positive patients showed better clinical characteristics than signature-negative patients

Each of the 1171 patients in the discovery cohort was classified as either relapse-free (signature-positive) or relapsing (signature-negative) by applying the “top-left” threshold (see “Methods”) on the predicted probability calculated by the four-SNP model. When compared with signature-negative patients, signature-positive patients had a 54–64% reduction in mean ARR (Table 4). Seventeen and 40% of signature-positive patients were able to maintain NEDA4 and NEDA3 status, respectively, for up to 12 months on Copaxone treatment. In contrast, a lower percentage, 12 and 32%, of the signature-negative patients were able to maintain NEDA4 and NEDA3 status on Copaxone treatment, respectively. Signature-positive patients had, on average, a longer time to first relapse (mean = 344.3 days, standard deviation (SD) = 57.06 days) when compared with signature-negative patients (mean = 316.9 days, SD = 92.10 days). In addition, signature-positive patients had fewer T1 lesions (mean = 1.63, SD = 4.25) and a lower volume of T2 lesions (mean = 14.18, SD = 15.92) at baseline when compared with signature-negative patients who had a higher number of T1 lesions (mean = 2.21, SD = 6.04) and a higher volume of T2 lesions (mean = 16.26, SD = 17.52) at baseline. EDSS was similar between signature-positive and signature-negative patients (mean = 2.48, SD = 1.21 and mean = 2.51, SD = 1.22, respectively). Overall, signature-positive patients showed consistent and clinically meaningful improvements over signature-negative patients across multiple clinical measurements that were not employed in developing the signature.

**Table 4:** Summary of ARR change based on predicted response

Cohort	Type of MS	Number of patients			Follow-up duration (years)	Mean ARR change: Sig + versus Sig−
		Total	Sig+	Sig−		
**Discovery**						
GALA DB	RRMS	639	323	316	1	−54%
FORTE DB		532	268	264	1	−64%
**Independent assessment**						
GALA OL	RRMS	333	190	143	~3	−14%
GA-9001 DB		38	21	17	~3	−13%
GA-9001 OL		74	35	39	~20	−22%
GA-9003 DB		40	21	19	0.75	−53%
GA-9003 OL		84	41	43	0.75	−49%
PreCISe DB	CIS	132	69	63	3	−5%
PreCISe OL		240	129	111	5	+14%
**Specificity assessment**						
BRAVO – Avonex	RRMS	310	176	134	2	+10%

Sig + (signature-positive) and sig − (signature-negative) indicate patients classified as relapse-free and relapsing, respectively, after applying the “top-left” threshold on the predicted probabilities from the four-SNP logistic regression model (see “Methods”). Mean ARR was calculated by dividing the total number of relapses in Sig + (or Sig−) patients by the total sum of exposure to Copaxone (in years). The difference between mean ARR of Sig + and Sig − patients is presented in the last column. *ARR* annualized relapse rate, *CIS* clinically isolated syndrome, *DB* double-blind phase, *OL* open-label phase, *RRMS* relapsing-remitting multiple sclerosis

### Independent assessment of the four-SNP signature

Table 5 shows the classification performance of the four-SNP signature in the discovery cohorts (AUC = 0.66) and in the independent assessment cohorts (AUC = 0.45 to 0.65). ARR reductions in signature-positive patients from the RRMS independent cohorts ranged from 13 to 53% (Table 4). CIS cohorts were inconclusive, with the signature-positive DB cohort showing a 5% lower ARR and the signature-positive OL cohort showing a 14% higher ARR compared to signature-negative patients. Additional file 6 plots the mean ARR change (signature-positive versus signature-negative) against the sample size of the cohort. The plot shows that the sample size of the cohorts is not a determinant of the mean ARR change. Additional file 7 summarizes the performance metrics of all of the three-SNP subsets. None of these models outperformed the four-SNP model. Additional file 8 shows the results of pairwise test of differences between the AUC in the discovery cohort relative to each of the independent cohorts.

**Table 5:** Model performance summary on all the cohorts

Cohort	Number of patients			Follow-up duration (years)	Specificity	Sensitivity	AUC
	Total	Sig+	Sig−				
**Discovery**							
GALA DB	639	323	316	1	66%	54%	0.65
FORTE DB	532	268	264	1	71%	54%	0.68
GALA DB + FORTE DB	1171	591	580	1	68%	54%	0.66
**Independent assessment**							
GALA OL	333	190	143	~3	47%	58%	0.54
GA-9001 DB	38	21	17	~3	41%	52%	0.45
GA-9001 OL	74	35	39	~20	48%	45%	0.49
GA-9003 DB	40	21	19	0.75	67%	61%	0.65
GA-9003 OL	84	41	43	0.75	67%	54%	0.59
PreCISe DB	132	69	63	3	48%	52%	0.49
PreCISe OL	240	129	111	5	49%	54%	0.50

Sig + (signature-positive) and Sig − (signature-negative) indicate patients classified as relapse-free and relapsing, respectively, after applying the “top-left” threshold on the predicted probabilities from the four-SNP logistic regression model (see “Methods”). AUC is a threshold-independent metric that computes the overall performance of the model at all possible thresholds on the predicted probabilities. All performance metrics are rounded to two decimal places.

*DB* double-blind phase, *OL* open-label phase

### The four-SNP signature was specific to Copaxone response and not to Avonex or to placebo

Signature-positive patients in the Avonex-treated arm of the BRAVO cohort did not show an ARR reduction. On the contrary, they showed an increase in ARR of 10% relative to signature-negative patients (Table 4). Furthermore, the four-SNP signature was not associated with response in the placebo-treated arm of the GALA DB cohort. Both these findings provided complementary evidence that the four-SNP signature is specific to Copaxone-response.

### Clinical characterization of patient subsets

To identify the subset of patients in whom the four-SNP signature was associated with clinically meaningful improvements in response, patients in the combined discovery cohort were split into five bins based on the quintiles of predicted probabilities generated by the four-SNP logistic regression model (Fig. 2). Patients in each bin were characterized using a set of relevant baseline and on-treatment clinical measures that were not used in the discovery of the four-SNP signature (Fig. 2b). Additional file 9 shows the results of a principal components analysis on all of these clinical response variables. The pattern of the principal component loadings indicated that the clinical response variables that were not used in building the four-SNP model were orthogonal to the ones that were used to train the model. Several of these alternative clinical measures showed, on average, steady improvements that corresponded with the quintiles of predicted probabilities from the four-SNP model. Patients in the bin with the highest predicted probabilities (0.90 to 0.94) of being relapse-free had the highest proportion of observed relapse-free Copaxone responders (95.3%), the highest mean group-level ARR reduction (93%), the longest mean time to first relapse (351.2 days), and the greatest percentage of patients who met the NEDA3 and NEDA4 definitions at 12 months after initiation of treatment (Fig. 2b). In contrast, the bin with the lowest predicted probabilities (0.26 to 0.81) of being relapse-free had the greatest proportion of Copaxone non-responders (29%), the lowest mean group-level ARR reduction (58%), the shortest time to first relapse (300.8 days), and the lowest percentage of patients who met the NEDA3 and NEDA4 definitions after initiation of treatment (Fig. 2b).

## Discussion

Inter-individual variability in patient response to each of the available therapies for multiple sclerosis, combined with the variable course of disease, emphasizes the need for tools that help guide treatment choice in multiple sclerosis. The current study on Copaxone, a first-line DMT with a well-established favorable efficacy and safety profile, constitutes the largest pharmacogenetic study in multiple sclerosis reported to date (Additional file 1). A four-SNP signature was identified as associated with treatment response. Signature-positive Copaxone-treated RRMS patients demonstrated better response in multiple clinically meaningful measures, including ARR, MRI, and NEDA in two discovery RRMS cohorts. Improved ARRs were also observed in five independent RRMS cohorts but not in either placebo- or Avonex-treated RRMS patients, demonstrating the predictive as opposed to prognostic nature of the signature, and its specificity to Copaxone. The signature identified a ~10% subset of Copaxone-treated RRMS patients with the highest clinical improvements.

Copaxone is a synthetic heterogeneous mixture of up to 10^29^ variant antigenic polymers [44]. Its MoA is complex [12–14] and not completely elucidated. Known mechanisms include suppression of autoimmune inflammatory processes by inducing type II monocytes, activation of HLA type I CD8+ T-cells, and an increase in the number of T-regulatory cells [14]. Copaxone protects the myelin sheath by competing with *MBP*, which it was designed to mimic [44]. It binds to HLA class II sites on APCs which present the antigen to naïve T-cells, resulting in the production of Copaxone-specific Th2 cells (Additional file 10). These cells migrate into the central nervous system, cross-react with MBP, and induce local secretion of anti-inflammatory cytokines [14]. Copaxone is also known to promote neurotrophic factors and induce B-cell activation [14, 45, 46]. Gene regions spanning the identified four-SNP signature, *HLA-DQB2*, *MBP*, *UVRAG*, and *ZAK(CDCA7)*, are known to be related to either the MoA of Copaxone or the pathophysiology of multiple sclerosis (Additional file 10): *HLA-DQB2* is involved in antigen processing and presentation, central to Copaxone’s MoA. Other *HLA* class II variants have been linked with response to Copaxone in prior candidate-gene studies [16–18, 22] but have not been reliably replicated. The HLA class II variant *DRB1* has been associated with treatment response to both IFN-β and Copaxone [47]. *MBP*, whose gene product is mimicked by Copaxone, has been shown to be associated with Copaxone response in at least one previous candidate-gene study [19]. Novel genetic associations identified in this study include *UVRAG* and *ZAK(CDCA7). UVRAG* was recently identified as a regulator of naïve peripheral T-cell homeostasis [48], and is in keeping with Copaxone’s effect on T-cells and with a previous candidate-gene study that reported an association between *TRB* and Copaxone response [17]. *ZAK*, a member of the *MAP3K* family, is known to be activated by stress and inflammation [49], while *CDCA7* variants are associated with cell division and brain lesion formation in multiple sclerosis [50]. Thus, the signature spans a multitude of mechanisms which are consistent with Copaxone’s complex MoA and are supported by gene-expression [13, 14] and physicochemical studies [51, 52]. Collectively, findings from the current study as well as other studies [13, 14, 51, 52] suggest that the association of the signature to treatment response is unique to Copaxone’s MoA, which depends on its physicochemical properties and distinguishes it from other glatiramoids and follow-on products.

Prior pharmacogenetic studies of Copaxone response have utilized candidate-gene approaches in cohorts largely drawn from observational and hospital-based patient populations [16–19, 21]. This has resulted in limited reproducibility, potentially due to variable response criteria and small sample sizes. In contrast, the current study assessed patient cohorts from two large phase III clinical trials in the discovery phase, with a combined sample size of 1171 patients. Subsequently, the identified four-SNP signature was assessed in five additional independent late-phase clinical trial cohorts with RRMS, two Copaxone-treated cohorts with CIS, as well as Avonex- and placebo-treated cohorts. Additionally, the study employed a comprehensive genome-wide SNP-chip with a coverage of around five million SNPs combined with a multi-step association analysis that selected SNPs with the maximum a priori evidence. This was followed by a Bayesian predictive modeling approach that systematically explored all possible SNP combinations and simultaneously evaluated the probability of inclusion of each of the SNPs in the signature. Adopting the Bayesian approach allowed efficient identification of a minimal set of SNPs with the greatest potential to generalize to newer populations and avoided the need for multiple hypotheses testing while reducing false discoveries.

Relapses are the primary target phenotype of DMTs in RRMS patients. Therefore, this study employed response definitions that incorporated relapses both for the initial identification of extreme-phenotypes of Copaxone response as well as for Bayesian modeling to identify the four-SNP signature. The presence of the signature was correlated with higher ARR reduction as well as increased time to first relapse even in patients who had been treated with Copaxone for up to 20 years. When compared with the 20-year cohorts, those with ~3 years of follow-up had a lower mean ARR reduction. However, it is challenging to interpret ARR patterns over time because they are dependent on several factors. For example, it is well known that RRMS patients eventually develop a secondary progressive type of disease which is accompanied by a decrease in ARR [53]. In enrichment clinical trial designs, patients with a higher baseline ARR experience a lower mean number of relapses as the duration of follow-up in the trial increases [54–56], a phenomenon termed “regression to the mean”. Both these factors result in a decrease in the absolute ARR with time in trial cohorts.

In comparison to signature-negative patients, signature-positive patients showed a larger reduction in ARR upon switching to Copaxone treatment in the OL phase in almost all of the trial cohorts studied (Additional file 11). Additionally, signature-positive patients showed better MRI parameters (T1 and T2 lesions), reflecting improvement in inflammatory disease activity and burden as well as increased NEDA3/4 that provide an overall assessment of disease progression. The consistent association of the signature with improvement in multiple clinical parameters, together with its specificity to Copaxone therapy versus placebo and Avonex, demonstrate its robustness.

Notwithstanding the comprehensive approach taken in this study, only a limited proportion of observed heterogeneity in response phenotypes could be explained by genetic variation. Specifically, the AUC, which quantifies the classification performance of the four-SNP signature in the overall Copaxone-treated population, ranged from 0.45 to 0.67, demonstrating insufficient discriminatory power for clinical practice. Nevertheless, the signature was able to identify a genetically homogeneous ~10% subset of the multiple sclerosis patient population in the discovery cohort with a 93% reduction in ARR versus baseline (Fig. 2) and substantially improved response in multiple clinical measures. In the four-SNP signature, *ZAK(CDCA7)* had a low MAF and imbalanced sample sizes in the patient groups with the major and minor allele (Additional file 3), which resulted in a wider confidence interval for its regression coefficient (and OR) (Table 3). However, the posterior inclusion probability for this SNP remained high (Additional file 5), indicating that the potential bias introduced by the imbalanced allelic groups had little impact on the identified signature.

It is also important to note that the clinical trial cohorts employed in the discovery of the signature had only a year of patient follow-up. In the context of a chronic disease such as multiple sclerosis that affects patients over several years of their lives, a year of follow-up might not be sufficient to observe consistent patterns of response to treatment. Analysis of additional clinically relevant response definitions in the context of the four-SNP signatures in Fig. 2 is a challenging task, given the diversity of response definitions employed by clinicians treating multiple sclerosis patients. Nevertheless, it is important to strive for consensus signatures and validate the performance of these signatures in genetically defined subsets of RRMS patients, in additional independent cohorts with larger sample sizes, and in non-Caucasian multiple sclerosis patients using a variety of clinical response definitions.

Studies examining the pharmacogenetics of response to IFN-β (Avonex) [57] share commonalities with this study in terms of both methodology, such as a multi-stage study design, and an emerging trend towards identifying multi-SNP signatures [22, 57]. Furthermore, genetic signatures detected in the IFN-β studies were specific to IFN-β and not generalizable to Copaxone [58], paralleling the Copaxone-specific nature of the four-SNP signature detected in this study. Interestingly, a recent assessment of the pharmacogenetics of IFN-β non-response identified genotypic patient subsets comprising ~17% of the cohort who were not likely to respond at all to IFN-β therapy [59]. These findings are analogous to the results in the current study, ﻿albeit we pursued identification of high-response rather than non-response. Overall, findings from pharmacogenetic studies on the two major DMTs in multiple sclerosis, Copaxone and IFN-β, demonstrate genetic associations that are DMT-specific but confined to a small subset of the RRMS population. This suggests that while genetics alone cannot fully account for drug response variability in the overall multiple sclerosis patient population, diagnostic tools that incorporate genetics or other factors that enable the definition of more homogeneous disease subtypes may aid in guiding treatment choices in multiple sclerosis.

## Conclusions

The findings from this study emphasize the need for rigorous, large-scale studies with multiple independent cohorts to fully understand the contribution of genetics to multiple sclerosis drug response. For the first time, a Copaxone-specific multi-SNP signature identifies patients with higher response to treatment in multiple, independent cohorts and over extended periods of treatment, lending more evidence to the contribution of genetic variation to drug response in patients with multiple sclerosis. The pronounced association of the signature with clinical improvements in a small subset of the patient cohort demonstrates the complex interplay of immune mechanisms and the individual nature of response to Copaxone.

## Additional files

Additional file 1: A comprehensive summary of sample sizes and cohorts in pharmacogenetics studies in the field of multiple sclerosis. (DOCX 23 kb)

Additional file 2: Candidate variants and genes from stage I of analysis. (DOCX 17 kb)

Additional file 3: Frequencies for each genotype of the four SNPs in the four-SNP model. (DOCX 14 kb)

Additional file 4: Notes on the model-building procedure used in the study. (DOCX 14 kb)

Additional file 5: The eleven-SNP model coefficients, odds ratios with 95% Bayesian confidence intervals and posterior inclusion probabilities. (DOCX 15 kb)

Additional file 6: Funnel-plot visualization of mean change in ARR (signature-positive versus signature-negative) versus the sample size of the cohort. Discovery as well as the independent cohorts are shown. (DOCX 58 kb)

Additional file 7: Performance metrics of each of the possible three-SNP models. (DOCX 16 kb)

Additional file 8: Results from the bootstrap version of the pairwise test of differences in AUC between the discovery cohort and each of the independent cohorts, originally described by Hanley and McNeil. (DOCX 15 kb)

Additional file 9: Principal component loadings of clinical response variables in discovery cohorts. (DOCX 118 kb)

Additional file 10: Biology of the four-SNP signature: A schematic illustration of the relationship of the identified four-SNP signature to the known components of Copaxone’s complex mechanism of action. (DOCX 1050 kb)

Additional file 11: Change in ARR among placebo patients who were switched to Copaxone treatment in the OL phase. (DOCX 12 kb)

Additional file 12: Details on participating institutional or clinical sites at which Institutional Review Boards or Ethics Committees approved the clinical trials included in the study. (DOCX 59 kb)

## Abbreviations

ARR: Annualized relapse rate
AUC: Area under the receiver operating characteristic curve
BRAVO: The Laquinimod DB placebo controlled study with a rater-Blinded Reference Arm of interferon β-1a (Avonex®)
BMA: Bayesian model averaging
CI: Confidence interval
CIS: Clinically isolated syndrome
DB: Double-blind
DMT: Disease-modifying therapy
EDSS: Expanded Disability Status Scale
FORTE: The FORTy mg Efficacy of glatiramer acetate study
GA: Glatiramer acetate
GALA: The Glatiramer Acetate Low-Frequency Administration study
HWE: Hardy–Weinberg equilibrium
IFN: Interferon
LD: Linkage disequilibrium
MBP: Myelin basic protein
MoA: Mechanism of action
MS: Multiple sclerosis
NEDA: No evidence of disease activity
OL: Open-label
PreCISe: The early glatiramer acetate in patients Presenting with a Clinically Isolated Syndrome study
ROC: Receiver operating characteristic
RRMS: Relapsing-remitting multiple sclerosis
SD: Standard deviation
SNP: Single nucleotide polymorphism
TRB: T-cell receptor beta
