# Supplementary material for: A pharmacogenetic signature of high response to Copaxone in late-phase clinical-trial cohorts of multiple sclerosis
Source: Genome Med. 2017 May 31;9:50. doi: 10.1186/s13073-017-0436-y (PMC5450152; doi:10.1186/s13073-017-0436-y)
Supplement: Supplementary file 1 — A comprehensive summary of sample sizes and cohorts in pharmacogenetics studies in the field of multiple sclerosis. (DOCX 23 kb) [file 13073_2017_436_MOESM1_ESM.docx]

| **Additional File 1: A comprehensive summary of sample sizes and cohorts in pharmacogenetics studies in the field of multiple sclerosis.** | | | | | | | |
| --- | --- | --- | --- | --- | --- | --- | --- |
| **Study** | **Year** | **Source of study population** | **Total number of patients assessed** | **Number of primary drug-treated patients assessed** | **Other disease-modifying therapies assessed** | **Number of patients on other disease modifying therapies** | **Number of placebo-treated patients assessed** |
| **A. Pharmacogenetics of Copaxone as primary drug** | | | | | | | |
| Fusco et al., 2001 [[1]](https://paperpile.com/c/uA3rnA/YSST1) | 2001 | Observational/Hospital-based | 83 | 44 | IFN-beta | 39 | - |
| Grossman *et al.*, 2007 [[2]](https://paperpile.com/c/uA3rnA/qQKc9) | 2007 | Clinical trial-based | 174 | 85 | - | - | 89 |
| Gross et al., 2011 [[3]](https://paperpile.com/c/uA3rnA/QPd8r) | 2011 | Observational/Hospital-based | 756 | 332 | IFN-beta | 424 | - |
| Tsareva et al., 2011 [[4]](https://paperpile.com/c/uA3rnA/e1kDH) | 2011 | Observational/Hospital-based | 285 | 285 | - | - | - |
| Dhib-Jalbut et al., 2013 [[5]](https://paperpile.com/c/uA3rnA/XmsbP) | 2013 | Observational/Hospital-based | 64 | 64 | - | - | - |
| Ross et al. (Current Study) | 2016 | Clinical trial-based | 2733 | 2112 (Discovery: 1171, Validation: 941) | IFN-beta | 310 | 311 |
| **B. Pharmacogenetics of IFN-beta as primary drug** | | | | | | | |
| Villoslada et al., 2002 [[6]](https://paperpile.com/c/uA3rnA/CbIeu) | 2002 | Observational/Hospital-based | 202 | 202 |  |  | - |
| Sriram et al., 2003 [[7]](https://paperpile.com/c/uA3rnA/E3e3D) | 2003 | Observational/Hospital-based | 147 | 147 |  |  | - |
| Cunningham et al., 2005 [[8]](https://paperpile.com/c/uA3rnA/o9G1Y)* | 2005 | Observational/Hospital-based | 230 | 230 |  |  | - |
| Fernández et al., 2005 [[9]](https://paperpile.com/c/uA3rnA/cUcOk) | 2005 | Observational/Hospital-based | 96 | 96 |  |  | - |
| Leyva et al., 2005 [[10]](https://paperpile.com/c/uA3rnA/dnofl)* | 2005 | Observational/Hospital-based | 147 | 147 |  |  | - |
| Wergeland et al., 2005 [[11]](https://paperpile.com/c/uA3rnA/mW7Pb) | 2005 | Clinical trial-based | 63 | 63 (IFN-beta: 38,  IFN-alpha: 25) |  |  | - |
| Martínez et al., 2006 [[12]](https://paperpile.com/c/uA3rnA/KZ7pp)* | 2006 | Observational/Hospital-based | 110 | 110 |  |  | - |
| Weinstock-Guttman et al., 2007 [[13]](https://paperpile.com/c/uA3rnA/l3J7) | 2007 | Observational/Hospital-based | 179 | 179 |  |  | - |
| Byun et al., 2008 [[14]](https://paperpile.com/c/uA3rnA/d6aAE) | 2008 | Observational/Hospital-based | 287 | 287 |  |  | - |
| Cénit et al., 2009 [[15]](https://paperpile.com/c/uA3rnA/HMuVo)* | 2009 | Observational/Hospital-based | 199 | 199 |  |  | - |
| O’Doherty et al., 2009 [[16]](https://paperpile.com/c/uA3rnA/gjT6Q) | 2009 | Observational/Hospital-based | 255 | 255 |  |  | - |
| Comabella et al., 2009 [[17]](https://paperpile.com/c/uA3rnA/8fIz) | 2009 | Observational/Hospital-based | 149 | 149 |  |  | - |
| Comabella et al., 2009[[18]](https://paperpile.com/c/uA3rnA/MJsN) | 2009 | Observational/Hospital-based | 200 | 200 (Discovery: 106, Validation: 94) |  |  | - |
| Vosslamber et al., 2011 [[19]](https://paperpile.com/c/uA3rnA/iK4w4) | 2011 | Observational/Hospital-based | 335 | 335 (Discovery: 75, Validation: 261) |  |  | - |
| Alvarez-Lafuente et al., 2011 [[20]](https://paperpile.com/c/uA3rnA/WPyxL)* | 2011 | Observational/Hospital-based | 163 | 163 |  |  | - |
| Malhotra et al., 2011 [[21]](https://paperpile.com/c/uA3rnA/4fi7z) | 2011 | Observational/Hospital-based | 281 | 281 |  |  | - |
| Vandenbroeck et al., 2011 [[22]](https://paperpile.com/c/uA3rnA/YkxZr) | 2011 | Observational/Hospital-based | 434 | 434 |  |  | - |
| Kulakova et al., 2012 [[23]](https://paperpile.com/c/uA3rnA/R8uBa) | 2012 | Observational/Hospital-based | 253 | 253 |  |  | - |
| Malhotra et al., 2013 [[24]](https://paperpile.com/c/uA3rnA/HBiL6) | 2013 | Observational/Hospital-based | 225 | 225 |  |  | - |
| López-Gómez et al., 2013 [[25]](https://paperpile.com/c/uA3rnA/NgZCF) | 2013 | Observational/Hospital-based | 812 | 735 (Discovery: 509, Validation: 226) | Copaxone | 77 | - |
| Torbati et al., 2015 [[26]](https://paperpile.com/c/uA3rnA/sugBq) | 2015 | Observational/Hospital-based | 120 | 120 |  |  | - |
| Esposito et al., 2015 [[27]](https://paperpile.com/c/uA3rnA/8Z39h) | 2015 | Observational/Hospital-based | 1343 | 997 (Discovery: 116, Validation: 881) | Copaxone | 346 | - |
| Mahurkar et al., 2016 [[28]](https://paperpile.com/c/uA3rnA/gHTCC) | 2016 | Observational/Hospital-based | 630 | 630 (Discovery: 151, Validation: 479) |  |  | - |
| **C. Pharmacogenetics of Mitoxantrone as primary drug** | | | | | | | |
| Cotte et al., 2009 [[29]](https://paperpile.com/c/uA3rnA/MuWt1) | 2009 | Observational/Hospital-based | 309 | 309 |  |  | - |
| Grey Née Cotte et al., 2015 [[30]](https://paperpile.com/c/uA3rnA/f22S4) | 2015 | Observational/Hospital-based | 239 | 196 |  |  | 43 |

Note: Studies marked with an asterisk also included healthy controls for purposes of assessing disease susceptibility, not pharmacogenetic response.

**References**

[1. Fusco C, Andreone V, Coppola G, Luongo V, Guerini F, Pace E, et al. HLA-DRB1*1501 and response to copolymer-1 therapy in relapsing-remitting multiple sclerosis. Neurology [Internet]. 2001;57:1976–9. Available from:](http://paperpile.com/b/uA3rnA/YSST1) <http://www.ncbi.nlm.nih.gov/pubmed/11739812>

[2. Grossman I, Avidan N, Singer C, Goldstaub D, Hayardeny L, Eyal E, et al. Pharmacogenetics of glatiramer acetate therapy for multiple sclerosis reveals drug-response markers. Pharmacogenet. Genomics [Internet]. 2007;17:657–66. Available from:](http://paperpile.com/b/uA3rnA/qQKc9) <http://dx.doi.org/10.1097/FPC.0b013e3281299169>

[3. Gross R, Healy BC, Cepok S, Chitnis T, Khoury SJ, Hemmer B, et al. Population structure and HLA DRB1 1501 in the response of subjects with multiple sclerosis to first-line treatments. J. Neuroimmunol. [Internet]. 2011;233:168–74. Available from:](http://paperpile.com/b/uA3rnA/QPd8r) <http://dx.doi.org/10.1016/j.jneuroim.2010.10.038>

[4. Tsareva EI, Kulakova OG, Makarycheva OI, Boĭko AN, Shchur SG, Lashch NI, et al. [Pharmacogenomics of multiple sclerosis: association of immune response genes polymorphism with copaxone treatment efficacy]. Mol. Biol. [Internet]. europepmc.org; 2011;45:963–72. Available from:](http://paperpile.com/b/uA3rnA/e1kDH) <http://www.ncbi.nlm.nih.gov/pubmed/22295566>

[5. Dhib-Jalbut S, Valenzuela RM, Ito K, Kaufman M, Ann Picone M, Buyske S. HLA DR and DQ alleles and haplotypes associated with clinical response to glatiramer acetate in multiple sclerosis. Mult. Scler. Relat. Disord. [Internet]. 2013;2:340–8. Available from:](http://paperpile.com/b/uA3rnA/XmsbP) <http://dx.doi.org/10.1016/j.msard.2013.02.005>

[6. Villoslada P, Barcellos LF, Rio J, Begovich AB, Tintore M, Sastre-Garriga J, et al. The HLA locus and multiple sclerosis in Spain. Role in disease susceptibility, clinical course and response to interferon-beta. J. Neuroimmunol. [Internet]. 2002;130:194–201. Available from:](http://paperpile.com/b/uA3rnA/CbIeu) <https://www.ncbi.nlm.nih.gov/pubmed/12225902>

[7. Sriram U, Barcellos LF, Villoslada P, Rio J, Baranzini SE, Caillier S, et al. Pharmacogenomic analysis of interferon receptor polymorphisms in multiple sclerosis. Genes Immun. [Internet]. nature.com; 2003;4:147–52. Available from:](http://paperpile.com/b/uA3rnA/E3e3D) <http://dx.doi.org/10.1038/sj.gene.6363946>

[8. Cunningham S, Graham C, Hutchinson M, Droogan A, O’Rourke K, Patterson C, et al. Pharmacogenomics of responsiveness to interferon IFN-beta treatment in multiple sclerosis: a genetic screen of 100 type I interferon-inducible genes. Clin. Pharmacol. Ther. [Internet]. 2005;78:635–46. Available from:](http://paperpile.com/b/uA3rnA/o9G1Y) <http://dx.doi.org/10.1016/j.clpt.2005.08.018>

[9. Fernández O, Fernández V, Mayorga C, Guerrero M, León A, Tamayo JA, et al. HLA class II and response to interferon-beta in multiple sclerosis. Acta Neurol. Scand. [Internet]. Munksgaard International Publishers; 2005;112:391–4. Available from:](http://paperpile.com/b/uA3rnA/cUcOk) <http://dx.doi.org/10.1111/j.1600-0404.2005.00415.x>

[10. Leyva L, Fernández O, Fedetz M, Blanco E, Fernández VE, Oliver B, et al. IFNAR1 and IFNAR2 polymorphisms confer susceptibility to multiple sclerosis but not to interferon-beta treatment response. J. Neuroimmunol. [Internet]. 2005;163:165–71. Available from:](http://paperpile.com/b/uA3rnA/dnofl) <http://dx.doi.org/10.1016/j.jneuroim.2005.02.010>

[11. Wergeland S, Beiske A, Nyland H, Hovdal H, Jensen D, Larsen JP, et al. IL-10 promoter haplotype influence on interferon treatment response in multiple sclerosis. Eur. J. Neurol. [Internet]. 2005;12:171–5. Available from:](http://paperpile.com/b/uA3rnA/mW7Pb) <http://dx.doi.org/10.1111/j.1468-1331.2004.01102.x>

[12. Martínez A, de las Heras V, Mas Fontao A, Bartolomé M, de la Concha EG, Urcelay E, et al. An IFNG polymorphism is associated with interferon-beta response in Spanish MS patients. J. Neuroimmunol. [Internet]. 2006;173:196–9. Available from:](http://paperpile.com/b/uA3rnA/KZ7pp) <http://dx.doi.org/10.1016/j.jneuroim.2005.12.002>

[13. Weinstock-Guttman B, Tamaño-Blanco M, Bhasi K, Zivadinov R, Ramanathan M. Pharmacogenetics of MXA SNPs in interferon-β treated multiple sclerosis patients. J. Neuroimmunol. [Internet]. 2007/1;182:236–9. Available from:](http://paperpile.com/b/uA3rnA/l3J7) <http://www.sciencedirect.com/science/article/pii/S0165572806003961>

[14. Byun E, Caillier SJ, Montalban X, Villoslada P, Fernández O, Brassat D, et al. Genome-wide pharmacogenomic analysis of the response to interferon beta therapy in multiple sclerosis. Arch. Neurol. [Internet]. 2008;65:337–44. Available from:](http://paperpile.com/b/uA3rnA/d6aAE) <http://dx.doi.org/10.1001/archneurol.2008.47>

[15. Cénit MDC, Blanco-Kelly F, de las Heras V, Bartolomé M, de la Concha EG, Urcelay E, et al. Glypican 5 is an interferon-beta response gene: a replication study. Mult. Scler. [Internet]. 2009;15:913–7. Available from:](http://paperpile.com/b/uA3rnA/HMuVo) <http://dx.doi.org/10.1177/1352458509106509>

[16. O’Doherty C, Favorov A, Heggarty S, Graham C, Favorova O, Ochs M, et al. Genetic polymorphisms, their allele combinations and IFN-beta treatment response in Irish multiple sclerosis patients. Pharmacogenomics [Internet]. 2009;10:1177–86. Available from:](http://paperpile.com/b/uA3rnA/gjT6Q) <http://dx.doi.org/10.2217/pgs.09.41>

[17. Comabella M, Fernández-Arquero M, Río J, Guinea A, Fernández M, Cenit MC, et al. HLA class I and II alleles and response to treatment with interferon-beta in relapsing-remitting multiple sclerosis. J. Neuroimmunol. [Internet]. 2009;210:116–9. Available from:](http://paperpile.com/b/uA3rnA/8fIz) <http://dx.doi.org/10.1016/j.jneuroim.2009.01.012>

[18. Comabella M, Craig DW, Morcillo-Suárez C, Río J, Navarro A, Fernández M, et al. Genome-wide scan of 500,000 single-nucleotide polymorphisms among responders and nonresponders to interferon beta therapy in multiple sclerosis. Arch. Neurol. [Internet]. 2009;66:972–8. Available from:](http://paperpile.com/b/uA3rnA/MJsN) <http://dx.doi.org/10.1001/archneurol.2009.150>

[19. Vosslamber S, van der Voort LF, van den Elskamp IJ, Heijmans R, Aubin C, Uitdehaag BMJ, et al. Interferon regulatory factor 5 gene variants and pharmacological and clinical outcome of Interferonβ therapy in multiple sclerosis. Genes Immun. [Internet]. 2011;12:466–72. Available from:](http://paperpile.com/b/uA3rnA/iK4w4) <http://dx.doi.org/10.1038/gene.2011.18>

[20. Alvarez-Lafuente R, Blanco-Kelly F, Garcia-Montojo M, Martínez A, De Las Heras V, Dominguez-Mozo MI, et al. CD46 in a Spanish cohort of multiple sclerosis patients: genetics, mRNA expression and response to interferon-beta treatment. Mult. Scler. [Internet]. 2011;17:513–20. Available from:](http://paperpile.com/b/uA3rnA/WPyxL) <http://dx.doi.org/10.1177/1352458510393263>

[21. Malhotra S, Morcillo-Suárez C, Brassat D, Goertsches R, Lechner-Scott J, Urcelay E, et al. IL28B polymorphisms are not associated with the response to interferon-beta in multiple sclerosis. J. Neuroimmunol. [Internet]. 2011;239:101–4. Available from:](http://paperpile.com/b/uA3rnA/4fi7z) <http://www.sciencedirect.com/science/article/pii/S0165572811002219>

[22. Vandenbroeck K, Alloza I, Swaminathan B, Antigüedad A, Otaegui D, Olascoaga J, et al. Validation of IRF5 as multiple sclerosis risk gene: putative role in interferon beta therapy and human herpes virus-6 infection. Genes Immun. [Internet]. 2011;12:40–5. Available from:](http://paperpile.com/b/uA3rnA/YkxZr) <http://dx.doi.org/10.1038/gene.2010.46>

[23. Kulakova OG, Tsareva EY, Boyko AN, Shchur SG, Gusev EI, Lvovs D, et al. Allelic combinations of immune-response genes as possible composite markers of IFN-β efficacy in multiple sclerosis patients. Pharmacogenomics [Internet]. Future Medicine; 2012;13:1689–700. Available from:](http://paperpile.com/b/uA3rnA/R8uBa) <http://dx.doi.org/10.2217/pgs.12.161>

[24. Malhotra S, Morcillo-Suárez C, Nurtdinov R, Rio J, Sarro E, Moreno M, et al. Roles of the ubiquitin peptidase USP18 in multiple sclerosis and the response to interferon-β treatment. Eur. J. Neurol. [Internet]. 2013;20:1390–7. Available from:](http://paperpile.com/b/uA3rnA/HBiL6) <http://dx.doi.org/10.1111/ene.12193>

[25. López-Gómez C, Pino-Ángeles A, Órpez-Zafra T, Pinto-Medel MJ, Oliver-Martos B, Ortega-Pinazo J, et al. Candidate Gene Study of TRAIL and TRAIL Receptors: Association with Response to Interferon Beta Therapy in Multiple Sclerosis Patients. PLoS One [Internet]. Public Library of Science; 2013 [cited 2016 Nov 21];8:e62540. Available from:](http://paperpile.com/b/uA3rnA/NgZCF) <http://journals.plos.org/plosone/article/file?id=10.1371/journal.pone.0062540&type=printable>

[26. Torbati S, Karami F, Ghaffarpour M, Zamani M. Association of CD58 Polymorphism with Multiple Sclerosis and Response to Interferon ß Therapy in A Subset of Iranian Population. Cell J. [Internet]. 2015;16:506–13. Available from:](http://paperpile.com/b/uA3rnA/sugBq) <https://www.ncbi.nlm.nih.gov/pubmed/25685741>

[27. Esposito F, Sorosina M, Ottoboni L, Lim ET, Replogle JM, Raj T, et al. A pharmacogenetic study implicates SLC9a9 in multiple sclerosis disease activity. Ann. Neurol. [Internet]. 2015;78:115–27. Available from:](http://paperpile.com/b/uA3rnA/8Z39h) <http://dx.doi.org/10.1002/ana.24429>

[28. Mahurkar S, Moldovan M, Suppiah V, Sorosina M, Clarelli F, Liberatore G, et al. Response to interferon-beta treatment in multiple sclerosis patients: a genome-wide association study. Pharmacogenomics J. [Internet]. nature.com; 2016; Available from:](http://paperpile.com/b/uA3rnA/gHTCC) <http://dx.doi.org/10.1038/tpj.2016.20>

[29. Cotte S, von Ahsen N, Kruse N, Huber B, Winkelmann A, Zettl UK, et al. ABC-transporter gene-polymorphisms are potential pharmacogenetic markers for mitoxantrone response in multiple sclerosis. Brain [Internet]. 2009;132:2517–30. Available from:](http://paperpile.com/b/uA3rnA/MuWt1) <http://dx.doi.org/10.1093/brain/awp164>

[30. Grey Née Cotte S, Salmen Née Stroet A, von Ahsen N, Starck M, Winkelmann A, Zettl UK, et al. Lack of efficacy of mitoxantrone in primary progressive Multiple Sclerosis irrespective of pharmacogenetic factors: a multi-center, retrospective analysis. J. Neuroimmunol. [Internet]. 2015;278:277–9. Available from:](http://paperpile.com/b/uA3rnA/f22S4) <http://dx.doi.org/10.1016/j.jneuroim.2014.11.017>
