# Supplementary material for: A pharmacogenetic signature of high response to Copaxone in late-phase clinical-trial cohorts of multiple sclerosis
Source: Genome Med. 2017 May 31;9:50. doi: 10.1186/s13073-017-0436-y (PMC5450152; doi:10.1186/s13073-017-0436-y)
Supplement: Supplementary file 2 — Candidate variants and genes from stage I of analysis. (DOCX 17 kb) [file 13073_2017_436_MOESM2_ESM.docx]

**Additional File 2: Candidate variants and genes from Stage I of analysis.**

| Table A2.1: The 35 prioritized candidate variants analyzed in Step 1 of Stage I analysis presented in Table 2 (main manuscript) | | | | |
| --- | --- | --- | --- | --- |
| Source | SNP rsID | Chromosome | Gene | Mutation |
| Grossman, 2007 | rs1415148 | 1 | CTSS |  |
| Grossman, 2007 | rs2275235 | 1 | CTSS |  |
| Grossman, 2007 | rs946685 | 1 | IL12RB2 |  |
| Patent US20150110733 | rs10931091 | 2 | AC074182.1 |  |
| Patent US20150110733 | rs17575455 | 2 | AC078940.2 |  |
| Patent US20150110733 | rs4344916 | 2 | AC083939.1 |  |
| Tsareva et al., 2011 | rs231775 | 2 | CTLA4 | Missense, T17A |
| Grossman, 2007 | rs1129055 | 3 | CD86 | Missense, A228 |
| Grossman, 2007 | rs2001791 | 3 | CD86 |  |
| Tsareva et al., 2011 | rs6897932 | 5 | IL7RA | Missense, T244I |
| Patent US20150110733 | rs3135388 | 6 | HLA-DRB1 | *15001 |
| Patent US20150110733 | rs3135391 | 6 | HLA-DRB1 | *1501,T118T |
| Patent US20150110733 | rs4148871 | 6 | TAP2 |  |
| Tsareva et al., 2011 | rs1800629 | 6 | TNF |  |
| Patent US20150110733 | rs10950359 | 7 | AC074389.1 |  |
| Patent US20150110733 | rs2521644 | 7 | NPY |  |
| Patent US20150110733 | rs1558896 | 7 | TAC1 |  |
| Patent US20150110733 | rs974060 | 7 | TAC1 |  |
| Patent US20150110733 | rs17771939 | 8 | AC016885.1 |  |
| Patent US20150110733 | rs10988087 | 9 | SET |  |
| Patent US20150110733 | rs947603 | 10 | CEP55 |  |
| Patent US20150110733 | rs12256889 | 10 | CYP26C1 |  |
| Patent US20150110733 | rs2487896 | 10 | HPSE2 |  |
| Patent US20150110733 | rs11599624 | 10 | P11-655H13 |  |
| Patent US20150110733 | rs4369324 | 10 | P11-655H13 |  |
| Patent US20150110733 | rs11617134 | 13 | P11-629E24 |  |
| Patent US20150110733 | rs1007328 | 15 | AC012409.1 |  |
| Patent US20150110733 | rs4343256 | 15 | CRTC3 |  |
| Patent US20150110733 | rs2177073 | 18 | DTNA |  |
| Patent US20150110733 | rs10853605 | 18 | MEX3C |  |
| Patent US20150110733 | rs9944913 | 18 | NOL4 |  |
| Patent US20150110733 | rs269976 | 18 | SLC14A2 |  |
| Patent US20150110733 | rs4890535 | 18 | SLC14A2 |  |
| Patent US20150110733 | rs6097801 | 20 | CYP24A1 |  |
| Patent US20150110733 | rs1573706 | 20 | PTPRT |  |

| Table A2.2: The 30 genes analyzed in Step 2 of Stage I analysis presented in Table 2 (main manuscript) | |
| --- | --- |
| Gene | Chromosome |
| CTSS | 1 |
| IL10 | 1 |
| IL12RB2 | 1 |
| CTLA4 | 2 |
| IL1R1 | 2 |
| CCR5 | 3 |
| DNAJC13 | 3 |
| ERAP2 | 5 |
| IL13 | 5 |
| Il3 | 5 |
| IL7R | 5 |
| HLA-DRB1 | 6 |
| TAP1 | 6 |
| TAP2 | 6 |
| TNF | 6 |
| NPY | 7 |
| TAC1 | 7 |
| TRB | 7 |
| AC016885.1 | 8 |
| ALOX5 | 10 |
| FAS | 10 |
| GRIK4 | 11 |
| CLIP | 12 |
| RRN3 | 16 |
| MBP | 18 |
| NFAT | 18 |
| NOL4 | 18 |
| SLC14A2 | 18 |
| IFNAR1 | 21 |
| FOXP3 | X |

**References:**

Grossman I, Avidan N, Singer C, Goldstaub D, Hayardeny L, Eyal E, et al. Pharmacogenetics of glatiramer acetate therapy for multiple sclerosis reveals drug-response markers. Pharmacogenet. Genomics [Internet]. 2007; 17:657–66. Available from: <http://dx.doi.org/10.1097/FPC.0b013e3281299169>

Tsareva EI, Kulakova OG, Makarycheva OI, Boĭko AN, Shchur SG, Lashch NI, et al. [Pharmacogenomics of multiple sclerosis: association of immune response genes polymorphism with copaxone treatment efficacy]. Mol. Biol. [Internet]. europepmc.org; 2011; 45:963–72. Available from: <http://www.ncbi.nlm.nih.gov/pubmed/22295566>

Tchelet A, Hayden M, Hayardeny L, Ross CJD, Grossman I, Ladkani D. Patent US20150110733: Genetic markers predictive of response to glatiramer acetate [Internet]. World Patent 2015[cited 2016 Jul 22] Available from: <https://www.google.com/patents/WO2015061367A1?cl=en>
