# Supplementary material for: A pharmacogenetic signature of high response to Copaxone in late-phase clinical-trial cohorts of multiple sclerosis
Source: Genome Med. 2017 May 31;9:50. doi: 10.1186/s13073-017-0436-y (PMC5450152; doi:10.1186/s13073-017-0436-y)
Supplement: Supplementary file 3 — Frequencies for each genotype of the four SNPs in the four-SNP model. (DOCX 14 kb) [file 13073_2017_436_MOESM3_ESM.docx]

| Additional File 3: Frequencies for each genotype of the 4 SNPs in the 4-SNP model | | | | | |
| --- | --- | --- | --- | --- | --- |
|  |  |  | **Genotypes*** | | |
| Gene | **Observed response status** | **SNP coding^** | **AA** | **AG** | **GG** |
| *UVRAG* | Non-responder (relapsing) | 0 | 144 | 0 | 0 |
|  |  | 1 | 0 | 43 | 0 |
|  |  | 2 | 0 | 0 | 1 |
|  | Responder (non-relapsing) | 0 | 863 | 0 | 0 |
|  |  | 1 | 0 | 118 | 0 |
|  |  | 2 | 0 | 0 | 2 |
| *HLA-DQB2* | Non-responder (relapsing) | 0 | 0 | 0 | 40 |
|  |  | 1 | 0 | 94 | 0 |
|  |  | 2 | 54 | 0 | 0 |
|  | Responder (non-relapsing) | 0 | 0 | 0 | 348 |
|  |  | 1 | 0 | 478 | 0 |
|  |  | 2 | 157 | 0 | 0 |
| *MBP* | Non-responder (relapsing) | 0 | 0 | 0 | 51 |
|  |  | 1 | 27 | 110 | 0 |
|  | Responder (non-relapsing) | 0 | 0 | 0 | 406 |
|  |  | 1 | 124 | 453 | 0 |
| *ZAK(CDCA7)* | Non-responder (relapsing) | 0 | 0 | 0 | 176 |
|  |  | 1 | 0 | 12 | 0 |
|  | Responder (non-relapsing) | 0 | 0 | 0 | 968 |
|  |  | 1 | 0 | 15 | 0 |

***Genotypes** correspond to the specific strand assayed on the array, based on Illumina’s method for consistent genotype calls (Please

see Illumina SNP Genotyping Technical Note at: https://www.illumina.com/documents/products/technotes/technote_topbot.pdf).

As a result, a given SNP with genotype AG can be interpreted as either AG or its complement, TC. ^**SNP coding** indicates the

inheritance model according to which the SNP was coded in the logistic regression model.
