# Supplementary material for: A pharmacogenetic signature of high response to Copaxone in late-phase clinical-trial cohorts of multiple sclerosis
Source: Genome Med. 2017 May 31;9:50. doi: 10.1186/s13073-017-0436-y (PMC5450152; doi:10.1186/s13073-017-0436-y)
Supplement: Supplementary file 4 — Notes on the model-building procedure used in the study. (DOCX 14 kb) [file 13073_2017_436_MOESM4_ESM.docx]

**Additional File 4: Notes on the model-building procedure used in the study.**

The logistic regression model we estimated using Bayesian Model Averaging (BMA) is an example of an ensemble-based parametric regression model [(Hoeting *et al.*, 1999)](https://paperpile.com/c/NIgtTb/Oita). We selected this Bayesian modeling method specifically for its capacity to perform better at simultaneous model selection and estimation in comparison to frequentist model selection methods [(Shankar *et al.*, 2015)](https://paperpile.com/c/NIgtTb/oyM3). Below we discuss the rationale of specifically selecting BMA as a parametric Bayesian regression method for selecting the SNP-signature over other comparable and widely-used frequentist variable selection algorithms such as logistic regression with LASSO and Elastic Net regularization as well as Random Forest.

Following the hypothesis generating phase in Stage I that identified the 11 SNPs, our goal was to identify the sparsest subset of the 11 SNPs that could yield nearly the same performance as the 11 SNPs. Thus, the resulting SNP signature would contain the subset of SNPs with the greatest predictive performance. For identifying this subset, we wanted to exhaustively assess all possible combinations (2^11^) of the 11-SNPs, while estimating a model for each combination in a manner that avoids overfitting and utilizes all samples from the original cohort. Because we chose a clinically relevant binary response definition, a natural model choice for determining this signature was logistic regression.

If we had chosen a frequentist approach (such as LASSO, Elastic net etc.) to select and estimate a model of a subset of 11 SNPs, such an approach would have required a two-step procedure [(Chernozhukov *et al.*, 2016)](https://paperpile.com/c/NIgtTb/oikn). The first step would have employed cross-validation to identify a subset of SNPs. The second step would have employed a bootstrap resampling procedure that uses multiple subsets of the training data to compute robust effect size estimates along with confidence intervals for each of the SNPs in the identified subset.

The cross-validation approach to estimating model parameters is known to be influenced by which observations are included in the training set and which in the validation set. An exhaustive leave-one-out-cross-validation (LOOCV) overcomes the limitations of limited k-fold cross-validation by including all data-points at least once in training and validation sets. However, LOOCV is known to result in higher variability of validation error [(James *et al.*, 2013)](https://paperpile.com/c/NIgtTb/u6c6).

Given these drawbacks, we chose BMA with a sparsity-promoting spike-and-slab prior [(George and Mcculloch, 1997)](https://paperpile.com/c/NIgtTb/8zMK) over frequentist approaches for simultaneous variable selection and model estimation. This was enabled by the use of Markov Chain Monte Carlo (MCMC) method for sampling the space of the 2^11^ possible SNP configurations while efficiently evaluating the likelihood of each configuration [(Scott and Varian, 2014)](https://paperpile.com/c/NIgtTb/0QnB). We ran 50K iterations of the MCMC which gave us a set of the most likely SNP signatures. The 50K models from the MCMC iterations enabled us to simultaneously compute the posterior inclusion probabilities of each of the SNPs as well as the 95% Bayesian confidence intervals of their regression coefficients. Unlike frequentist resampling procedures, BMA uses the entire cohort for estimation and avoids the need for multi-step estimation. Using sparsity-promoting priors, a large number of MCMC samples (which gives rise to a large ensemble) and the full original cohort size for estimation have been shown to provide substantial protection against model over-fitting [(Castillo *et al.*, 2015)](https://paperpile.com/c/NIgtTb/w6Nq). Indeed, given these characteristics, prior work has found that BMA outperforms frequentist ensembles in simulation settings [(Shankar *et al.*, 2015)](https://paperpile.com/c/NIgtTb/oyM3).

**References**

[Castillo I, Schmidt-Hieber J, van der Vaart A. Bayesian linear regression with sparse priors [Internet]. Ann. Stat. 2015; 43: 1986–2018.[cited 2015 Nov 8] Available from:](http://paperpile.com/b/NIgtTb/w6Nq) <http://projecteuclid.org/download/pdfview_1/euclid.aos/1438606851>

[Chernozhukov V, Chetverikov D, Demirer M, Duflo E, Hansen C, Newey AW. Double Machine Learning for Treatment and Causal Parameters [Internet]. arXiv [stat.ML] 2016Available from:](http://paperpile.com/b/NIgtTb/oikn) <http://arxiv.org/abs/1608.00060>

[George EI, Mcculloch RE. Approaches for Bayesian variable selection [Internet]. In: Statistica Sinica. 1997. p. 339–373.[cited 2014 Jun 2] Available from:](http://paperpile.com/b/NIgtTb/8zMK) <http://citeseerx.ist.psu.edu/viewdoc/summary?doi=10.1.1.211.4871>

[Hoeting JA, Madigan D, Raftery AE, Volinsky CT. Bayesian model averaging: a tutorial (with comments by M. Clyde, David Draper and E. I. George, and a rejoinder by the authors) [Internet]. Stat. Sci. 1999; 14: 382–417.[cited 2014 Jun 2] Available from:](http://paperpile.com/b/NIgtTb/Oita) <http://projecteuclid.org/euclid.ss/1009212519>

[James G, Witten D, Hastie T, Tibshirani R. An Introduction to Statistical Learning: with Applications in R (Springer Texts in Statistics) [Internet]. 2013th ed. Springer Science & Business; 2013. Available from:](http://paperpile.com/b/NIgtTb/u6c6) <http://dx.doi.org/10.1007/978-1-4614-7138-7>

[Scott SL, Varian HR. Predicting the present with Bayesian structural time series [Internet]. IJMMNO 2014; 5: 4.[cited 2014 Jun 22] Available from:](http://paperpile.com/b/NIgtTb/0QnB) <http://www.inderscience.com/link.php?id=59942>

[Shankar J, Szpakowski S, Solis NV, Mounaud S, Liu H, Losada L, et al. A systematic evaluation of high-dimensional, ensemble-based regression for exploring large model spaces in microbiome analyses [Internet]. BMC Bioinformatics 2015; 16: 31.Available from:](http://paperpile.com/b/NIgtTb/oyM3) <http://dx.doi.org/10.1186/s12859-015-0467-6>
