# Supplementary material for: A pharmacogenetic signature of high response to Copaxone in late-phase clinical-trial cohorts of multiple sclerosis
Source: Genome Med. 2017 May 31;9:50. doi: 10.1186/s13073-017-0436-y (PMC5450152; doi:10.1186/s13073-017-0436-y)
Supplement: Supplementary file 5 — The eleven-SNP model coefficients, odds ratios with 95% Bayesian confidence intervals and posterior inclusion probabilities. (DOCX 15 kb) [file 13073_2017_436_MOESM5_ESM.docx]

**Additional File 5:** The eleven-SNP model coefficients and odds ratios with 95% Bayesian confidence intervals and posterior inclusion probabilities.

| SNP rsID | Gene | Regression coefficient (95% CI) | Odds Ratio (95% CI) | PIP |
| --- | --- | --- | --- | --- |
| *rs80191572* | *UVRAG* | -0.73 (-1.12, -0.32) | 0.48 (0.33, 0.73) | 91.70% |
| *rs28724893* | *HLA-DQB2/DOB* | -0.48 (-0.72, -0.23) | 0.62 (0.48, 0.80) | 95.80% |
| *rs1789084* | *MBP* | -0.63 (-1.00, -0.26) | 0.53 (0.37, 0.77) | 95.10% |
| *rs139890339* | *ZAK(CDCA7)* | -1.45 (-2.26, -0.60) | 0.24 (0.10, 0.55) | 86.50% |
| *rs117602254* | *PTPRT* | -0.91 (-1.59, -0.21) | 0.40 (0.20, 0.81) | 40.40% |
| *rs759458* | *SLC1A4* | 0.27 (-0.00, 0.55) | 1.31 (1.00, 1.73) | 19.00% |
| *rs16886004* | *MAGI2* | 0.36 (-0.01, 0.74) | 1.43 (0.99, 2.09) | 18.90% |
| *rs10162089* | *ALOX5AP* | 0.27 (-0.10, 0.60) | 1.30 (0.91, 1.83) | 13.50% |
| *rs1894408* | *HLA-DOB/TAP2* | 0.29 (-0.10, 0.70) | 1.34 (0.90, 2.02) | 13.20% |
| *rs73166319* | *SLC5A4/RFPL3* | 0.51(-0.25, 1.43) | 1.66 (0.78, 4.16) | 6.80% |
| *rs3135391* | *HLA-DRB1*15:01* | -0.04 (-0.46, 0.33) | 0.97 (0.63, 1.39) | 4.60% |

Coefficients of the eleven-SNP model were obtained by fitting a logistic regression model on data from the patients treated with Copaxone in the GALA DB and FORTE DB studies. SNPs from *MBP, MAGI2, ALOX5AP, HLA-DOB/TAP2 and HLA-DRB1*15:01* were coded according to a dominant inheritance model. All the other SNPs were coded according to an additive inheritance model. The logistic regression model estimated the log odds of being relapse-free conditional on the four SNPs. A negative regression coefficient for a given SNP implies that the major allele (coded as the reference level in the logistic regression model) was associated with increased odds of being relapse-free while the minor allele was associated with increased odds of relapses. The logistic regression model was estimated using Bayesian Model Averaging with an MCMC method that sampled the space of the 2^11^ possible SNP combinations with 50,000 models. The posterior inclusion probability (PIP) of each SNP indicates the percentage of the models where a given SNP was selected. The top four SNPs in genes, *UVRAG*, *HLA-DQB2/DOB, MBP* and *ZAK(CDCA7)*, were selected in the largest number of models (both with and without the baseline clinical covariates in the model). The rest of the SNPs were selected in a substantially lower number of models, as indicated by their relatively low PIPs. In addition, other than the top four SNPs, none of the SNPs had significant coefficients (as indicated by their 95% Bayesian confidence intervals) in both the models with and without the baseline clinical covariates.
