# Supplementary material for: A pharmacogenetic signature of high response to Copaxone in late-phase clinical-trial cohorts of multiple sclerosis
Source: Genome Med. 2017 May 31;9:50. doi: 10.1186/s13073-017-0436-y (PMC5450152; doi:10.1186/s13073-017-0436-y)
Supplement: Supplementary file 6 — Funnel-plot visualization of mean change in ARR (signature-positive versus signature-negative) versus the sample size of the cohort. Discovery as well as the independent cohorts are shown. (DOCX 58 kb) [file 13073_2017_436_MOESM6_ESM.docx]

**Additional File 6: Funnel-plot visualization of mean change in ARR (Sig+ vs. Sig-) vs.**

**the sample size of the cohort (discovery as well as the independent cohorts are shown).**

The funnel-plot shows that the sample size of the cohorts is not a determinant of

the mean ARR-change.
