# Supplementary material for: A pharmacogenetic signature of high response to Copaxone in late-phase clinical-trial cohorts of multiple sclerosis
Source: Genome Med. 2017 May 31;9:50. doi: 10.1186/s13073-017-0436-y (PMC5450152; doi:10.1186/s13073-017-0436-y)
Supplement: Supplementary file 7 — Performance metrics of each of the possible three-SNP models. (DOCX 16 kb) [file 13073_2017_436_MOESM7_ESM.docx]

| Additional File 7: Performance metrics of each of the possible three-SNP models. | | | | | | | | |
| --- | --- | --- | --- | --- | --- | --- | --- | --- |
| SNPs in model | Cohort | Total | Sig+ | Sig- | Follow-up duration (years) | Specificity | Sensitivity | AUC |
| HLA-DQB2, MBP, ZAK(CDCA7) | GALA DB | 639 | 364 | 275 | 1 | 0.58 | 0.6 | 0.63 |
| HLA-DQB2, MBP, ZAK(CDCA7) | FORTE DB | 532 | 296 | 236 | 1 | 0.62 | 0.58 | 0.66 |
|  |  |  |  |  |  |  |  |  |
| UVRAG, HLA-DQB2, MBP | GALA DB | 639 | 331 | 308 | 1 | 0.65 | 0.55 | 0.63 |
| UVRAG, HLA-DQB2, MBP | FORTE DB | 532 | 273 | 259 | 1 | 0.69 | 0.55 | 0.68 |
|  |  |  |  |  |  |  |  |  |
| UVRAG, HLA-DQB2, ZAK(CDCA7) | GALA DB | 639 | 450 | 189 | 1 | 0.46 | 0.74 | 0.62 |
| UVRAG, HLA-DQB2, ZAK(CDCA7) | FORTE DB | 532 | 363 | 169 | 1 | 0.51 | 0.71 | 0.67 |
|  |  |  |  |  |  |  |  |  |
| UVRAG, MBP, ZAK(CDCA7) | GALA DB | 639 | 209 | 430 | 1 | 0.78 | 0.35 | 0.6 |
| UVRAG, MBP, ZAK(CDCA7) | FORTE DB | 532 | 179 | 353 | 1 | 0.82 | 0.36 | 0.64 |

To classify patients as either relapse-free or relapsing, an optimal threshold on the predicted probabilities from the multi-SNP logistic regression model was determined. This threshold maximizes the sensitivity and specificity of the signature and corresponds to the point on the ROC curve closest to the top left corner (“top-left" threshold). Sig+ (Signature-positive) patients were those who either met or exceeded the predicted probability that corresponded to the “top-left” threshold in the multi-SNP model. The AUC is a threshold-independent metric that computes the overall performance of the model at all possible thresholds on the predicted probabilities.
