# Supplementary material for: A pharmacogenetic signature of high response to Copaxone in late-phase clinical-trial cohorts of multiple sclerosis
Source: Genome Med. 2017 May 31;9:50. doi: 10.1186/s13073-017-0436-y (PMC5450152; doi:10.1186/s13073-017-0436-y)
Supplement: Supplementary file 8 — Results from the bootstrap version of the pairwise test of differences in AUC between the discovery cohort and each of the independent cohorts, originally described by Hanley and McNeil. (DOCX 15 kb) [file 13073_2017_436_MOESM8_ESM.docx]

**Additional File 8: Results from the bootstrap pairwise test of differences in AUC between the discovery cohort (GALA DB + FORTE DB, AUC = 0.66 (see Table 5)) and each of the independent cohorts.**

| Cohort | Total number of patients | AUC for 4-SNP signature | P-value (vs. discovery AUC) |
| --- | --- | --- | --- |
| Discovery | | | |
| GALA DB + FORTE DB | 1171 | 0.66 | Reference |
| Independent Assessment | | | |
| GALA OL | 333 | 0.54 | 0.0137 |
| GA-9001 DB | 38 | 0.45 | 0.0313 |
| GA-9001 OL | 74 | 0.49 | 0.0566 |
| GA-9003 DB | 40 | 0.65 | 0.9823 |
| GA-9003 OL | 84 | 0.59 | 0.3384 |
| PreCISe DB | 132 | 0.49 | 0.0145 |
| PreCISe OL | 240 | 0.5 | 0.0042 |

AUC: area under the ROC curve. DB: double-blind phase. OL: open-label phase. The bootstrap test was originally described by Hanley and McNeil, and implemented in the pROC R package [1].

**References**

1. Robin X, Turck N, Hainard A, Tiberti N, Lisacek F, Sanchez J-C, et al. pROC: an open-source package for R and S+ to analyze and compare ROC curves. BMC Bioinformatics. 2011; 12:77. Available from: http://dx.doi.org/10.1186/1471-2105-12-77
