# Supplementary material for: A pharmacogenetic signature of high response to Copaxone in late-phase clinical-trial cohorts of multiple sclerosis
Source: Genome Med. 2017 May 31;9:50. doi: 10.1186/s13073-017-0436-y (PMC5450152; doi:10.1186/s13073-017-0436-y)
Supplement: Supplementary file 9 — Principal component loadings of clinical response variables in discovery cohorts. (DOCX 118 kb) [file 13073_2017_436_MOESM9_ESM.docx]

**Additional File 9: Principal component loadings of clinical response**

**variables in discovery cohorts.**

The top-five principal component loadings that cumulatively explained

~90% of the variance are shown. The pattern of the loadings indicated

that the clinical response variables that were not used in building the

model were orthogonal to the ones that were used to train the 4-SNP

model. Time-to-relapse, the binary relapse-free response variable,

on-trial ARR as well as the change in ARR had the largest loadings

on the first principal component while MRI variables and EDSS variables

loaded on the other principal components.
