# Supplementary material for: A pharmacogenetic signature of high response to Copaxone in late-phase clinical-trial cohorts of multiple sclerosis
Source: Genome Med. 2017 May 31;9:50. doi: 10.1186/s13073-017-0436-y (PMC5450152; doi:10.1186/s13073-017-0436-y)
Supplement: Supplementary file 10 — Biology of the four-SNP signature: A schematic illustration of the relationship of the identified four-SNP signature to the known components of Copaxone’s complex mechanism of action. (DOCX 1050 kb) [file 13073_2017_436_MOESM10_ESM.docx]

** Additional File 10: Biology of the four-SNP signature: A schematic illustration**

**of the relationship of the identified four-SNP signature to the known**

**components of Copaxone’s complex mechanism of action.**
