# Supplementary material for: A pharmacogenetic signature of high response to Copaxone in late-phase clinical-trial cohorts of multiple sclerosis
Source: Genome Med. 2017 May 31;9:50. doi: 10.1186/s13073-017-0436-y (PMC5450152; doi:10.1186/s13073-017-0436-y)
Supplement: Supplementary file 11 — Change in ARR among placebo patients who were switched to Copaxone treatment in the OL phase. (DOCX 12 kb) [file 13073_2017_436_MOESM11_ESM.docx]

| Cohort | Type of MS | 4-SNP signature status | Total | Difference in mean ARR: OL phase (on treatment) vs DB phase (on placebo) |
| --- | --- | --- | --- | --- |
| 9001 | RRMS | Negative | 22 | -78% |
|  |  | Positive | 15 | -70% |
| 9003 |  | Negative | 27 | -40% |
|  |  | Positive | 21 | -70% |
| GALA |  | Negative | 147 | -51% |
|  |  | Positive | 196 | -58% |
| PreCISe | CIS | Negative | 53 | -49% |
|  |  | Positive | 66 | -58% |

**Additional File 11: Change in ARR among placebo patients who were switched to Copaxone treatment in the OL phase.**
