# Supplementary material for: A pharmacogenetic signature of high response to Copaxone in late-phase clinical-trial cohorts of multiple sclerosis
Source: Genome Med. 2017 May 31;9:50. doi: 10.1186/s13073-017-0436-y (PMC5450152; doi:10.1186/s13073-017-0436-y)
Supplement: Supplementary file 12 — Details on participating institutional or clinical sites at which Institutional Review Boards or Ethics Committees approved the clinical trials included in the study. (DOCX 59 kb) [file 13073_2017_436_MOESM12_ESM.docx]

**Additional File 12: Participating Institutional or Clinical sites at which Institutional Review Boards or Ethics Committees approved the clinical trials included in the study.**

| GA-9001 DB (ClinicalTrials.gov: NCT00004814) and GA-9001 OL (ClinicalTrials.gov: NCT00203021) | | |
| --- | --- | --- |
| Primary Publications: | Johnson KP, Brooks BR, Cohen JA, Ford CC, Goldstein J, Lisak RP, et al. Copolymer 1 reduces relapse rate and improves disability in relapsing-remitting multiple sclerosis: results of a phase III multicenter, double-blind placebo-controlled trial. The Copolymer 1 Multiple Sclerosis Study Group. Neurology. 1995;45:1268–76. Available from: <http://www.ncbi.nlm.nih.gov/pubmed/7617181>  Johnson KP, Brooks BR, Cohen JA, Ford CC, Goldstein J, Lisak RP, et al. Extended use of glatiramer acetate (Copaxone) is well tolerated and maintains its clinical effect on multiple sclerosis relapse rate and degree of disability. Copolymer 1 Multiple Sclerosis Study Group. Neurology. 1998;50:701–8. Available from: http://www.ncbi.nlm.nih.gov/pubmed/9521260 | |
| Ethics Statements | The protocol was approved by the institutional review boards of the participating clinical centers, and all patients gave written informed consent (Johnson et al., 1995).  All patients signed a new informed consent statement before continuation in the extension phase (Johnson et al. 1998) | |
| Site Number | **Country** | **Participating Institution at which Ethics Committees or Institutional Review Boards approved the clinical trial** |
|  | USA | University of Pennsylvania Medical Center |
|  | USA | University of New Mexico School of Medicine |
|  | USA | Wayne State University Health Center |
|  | USA | UCLA School of Medicine |
|  | USA | University of Maryland |
|  | USA | University of Utah – VA Medical Center |
|  | USA | University of Rochester |
|  | USA | Yale University School of Medicine |
|  | USA | University of Southern California |
|  | USA | University of Texas – Health Science Center |
|  | USA | University of Wisconsin Hospital and Clinic |

| GALA DB and GALA OL (ClinicalTrials.gov: NCT01067521) | | |
| --- | --- | --- |
| Primary Publications: | Khan O, Rieckmann P, Boyko A, Selmaj K, Zivadinov R, GALA Study Group. Three times weekly glatiramer acetate in relapsing-remitting multiple sclerosis. Ann. Neurol. 2013;73:705–13. Available from: http://dx.doi.org/10.1002/ana.23938  Khan O, Rieckmann P, Boyko A, Selmaj K, Ashtamker N, Davis MD, et al. Efficacy and safety of a three-times-weekly dosing regimen of glatiramer acetate in relapsing-remitting multiple sclerosis patients: 3-year results of the Glatiramer Acetate Low-Frequency Administration open-label extension study. Mult. Scler. 2016;1352458516664033. Available from: http://dx.doi.org/10.1177/1352458516664033 | |
| Ethics Statements | All institutional review boards or ethics committees of the participating centers approved the protocol, and all patients gave written informed consent before any study-related procedures were performed. Study progress was overseen by an independent data-monitoring committee. (Khan et al., 2013).  All institutional review boards or ethical committees of the participating centers approved the protocol, and all patients gave written informed consent before any study-related procedures were performed. (Khan et al. 2016) | |
| Site Number | **Country** | **Participating Institution at which Ethics Committees or Institutional Review Boards approved the clinical trial** |
|  | Bulgaria | 1st MHAT, Sofia |
|  | Bulgaria | CCB Medical Institute - Ministry of Interiority |
|  | Bulgaria | MHAT Dr. Stefan Cherkezov, Department of Neurology |
|  | Bulgaria | MHAT Sveti Georgi |
|  | Bulgaria | MHAT Trakia EOOD |
|  | Bulgaria | MHAT Puls |
|  | Bulgaria | MHAT Ruse, 1st Department of Neurology |
|  | Bulgaria | MHAT Shumen, Neurology Department |
|  | Bulgaria | MHAT Tokuda Hospital Sofia AD, Department of Neurology |
|  | Bulgaria | MMA HAT Sofia - Clinic of Functional Diagnostics of Nervous System |
|  | Bulgaria | MMA HAT Sofia - Clinic of Neurology |
|  | Bulgaria | MHAT Sveta Marina EAD |
|  | Bulgaria | SHATNPsy - St. Naum EAD |
|  | Bulgaria | UMHAT Dr. Georgi Stranski |
|  | Bulgaria | UMHAT St. Ivan Rilski EAD |
|  | Bulgaria | UMHAT Tsaritsa Yoanna |
|  | Bulgaria | UMHAT Alexandrovska EAD |
|  | Bulgaria | UMHAT Pleven - First Clinic of Neurology |
|  | Croatia | Clinical Hospital Center Zagreb |
|  | Croatia | Clinical Hospital Osijek |
|  | Croatia | Clinical Hospital Dubrava |
|  | Croatia | Clinical Hospital Sveti Duh |
|  | Czech Republic | Faculty Hospital Olomouc Department of Neurology |
|  | Czech Republic | Faculty Hospital Ostrava, Department of Neurology |
|  | Czech Republic | Hospital Teplice, Neurology department |
|  | Estonia | East Tallinn Central Hospital - Department of Neurology |
|  | Georgia | LTD Petre Sarajishvili Institute of Neurology |
|  | Georgia | Ltd Medical Center CITO |
|  | Germany | NeuroCure Clinical Research Center |
|  | Germany | Universitaet Ulm - Neurology Department |
|  | Germany | Technische Universitaet Dresden - Neurologische Universitaetsklinik |
|  | Germany | Neurologisches Reha-Zentrum Quellenhof Bad Wildbad GmbH |
|  | Germany | Neurologische Praxis Alexander Simonow |
|  | Germany | Krankenhaus Hohe Warte Bayreuth GmbH |
|  | Germany | Universitaetsklinikum Klinik fur Nervenheilkunde Abteilung Neurologie |
|  | Germany | Charite Campus Benjamin Franklin, Klinik und Hochschulambulanz fuer Neurologie |
|  | Hungary | Szent Imre Hospital, Department of Neurology |
|  | Hungary | Kenezy Hospital and Polyclinic, Department of Neurology |
|  | Hungary | Markhot Ferenc Hospital, Department of Neurology |
|  | Hungary | Petz Aladar Teaching Hospital, Department of Neurology |
|  | Hungary | Vaszary Kolos Hospital, Department of Neurology |
|  | Israel | Sheba Medical Center-MS Center |
|  | Italy | IFO Istituto Nazionale Regina Elena - Struttura Semplice Dipartimentale di Neurologia |
|  | Italy | Ospedale Bellaria - Azienda USL di Bologna |
|  | Lithuania | Hospital of Lithuanian University of Health Sciences Kaunas Clinics |
|  | Lithuania | Vilnius University Hospital Santariski Klinikos, Centre of Neurology |
|  | Lithuania | Republican Siauliai Hospital |
|  | Poland | SPZOZ Uniwersytecki Szpital Kliniczny Nr 1 im. Norberta Barlickiego UM w Lodzi |
|  | Poland | SP Centralny Szpital Kliniczny |
|  | Poland | NZOZ Neuro-Medic |
|  | Poland | SP ZOZ Szpital Specjalistyczny w Koscierzynie, Oddzial Neurologiczny |
|  | Poland | Klinika Neurologii, Akademicki Szpital Kliniczny im. Jana Mikulicza-Radeckiego we Wroclawiu |
|  | Poland | Centrum Medyczne Euromedis |
|  | Poland | Samodzielny Publiczny Specjalistyczny Szpital Zachodni im. Jana Pawla II |
|  | Poland | SPZOZ Wojewodzki Szpital Specjalistyczny w Olsztynie |
|  | Poland | Wojewodzki Szpital Specjalistyczny im. NMP |
|  | Poland | NZOZ Centrum Medyczne DENDRYT |
|  | Poland | NZOZ Przychodnia Neurologiczna Belon, Krynicka, Sobkowiak-Osinska s.c. |
|  | Poland | Centrum Kliniczno Badawcze s.c. Specjalistyczna Grupowa Praktyka Lekarska |
|  | Poland | Szpital Specjalistyczny im. L. Rydygiera w Krakowie |
|  | Poland | RESMEDICA Elzbieta Jasinska |
|  | Poland | Pomorskie Centrum Traumatologii, Wojewodzki Szpital Specjalistyczny im. M. Kopernika, Oddz. Neuro. |
|  | Poland | Indywidualna Specjalistyczna Praktyka Lekarska |
|  | Poland | Zaklad Opieki Zdrowotnej, Specjalistyczny Szpital Sw. Lukasza |
|  | Poland | Szpital Kliniczny im. Heliodora Swiecickiego UM im. Karola Marcinkowskiego w Poznaniu |
|  | Romania | University Emergency Hospital Bucharest, Neurology Department |
|  | Romania | Fundeni Clinical Hospital, Neurology Department |
|  | Romania | County Emergency Hospital Piatra-Neamt |
|  | Romania | County Clinical Hospital Timisoara, Neurology Department |
|  | Romania | CFR University Hospital Constanta, Neurology Department |
|  | Romania | Constanta County Clinical Emergency Hospital, Neurology Department |
|  | Romania | Cluj County Clinical Emergency Hospital, Radiology and Medical Imaging Department |
|  | Romania | County Clinical Emergency Hospital Sibiu |
|  | Romania | County Clinical Hospital Targu Mures, Neurology Department |
|  | Romania | Clinical Rehabilitation Hospital Cluj, Neurology Department |
|  | Romania | Iasi Clinical Rehabilitation Hospital, Neurology Department |
|  | Romania | Neuropsychiatry Clinical Hospital Craiova, Neurology Department |
|  | Romania | Prof. Dr. Agrippa Ionescu Clinical Emergency Hospital |
|  | Russia | Ltd Medical Center CITO |
|  | Russia | State Budgetary Healthcare Institution City Clinical Hospital #24 of Healthcare Dep. of Moscow |
|  | Russia | Institution of Russian Academy of Science Human Brain Institute of Russian Academy of Science |
|  | Russia | Saint Petersburg State Healthcare Institution Municipal Multifield Hospital #2 |
|  | Russia | Military Medical Academy n.a. S. M. Kirov, Neurology Hospital |
|  | Russia | State Healthcare Institution Nizhny Novgorod N.A. Semashko Regional Clinical Hospital |
|  | Russia | Samarskaya Regional Hospital n.a. M. I. Kalinin |
|  | Russia | Sverdlovskaya Regional Clinical Hospital #1-Neurology Department |
|  | Russia | Perm State Medical Academy - Department of Neurology |
|  | Russia | Bashkir State Medical University of Roszdrav |
|  | Russia | Novosibirsk State Regional Hospital, Center of MS |
|  | Russia | State Healthcare Institution Territorial Clinical Hospital |
|  | Russia | Smolensk State Medical Academy, Neurology Department |
|  | Russia | Municipal Clinical Hospital #8 |
|  | Russia | Siberian State Medical University of Roszdrav |
|  | Russia | Krasnoyarsk State Medical University |
|  | Russia | Irkutsk State Institute for Advanced Medical Education of Roszdrav |
|  | Russia | S.I. Georgiyevsky Crimean Medical University, Chair of Nervous Diseases; Neurology Department |
|  | South Africa | Willows Medical Centre, Neurology |
|  | South Africa | Rosebank Medical and Dental Centre, Neurology |
|  | Ukraine | Lviv National Medical University, Chair of Neurology, Lviv Regional Clinical Hospital, Neurology Dep |
|  | Ukraine | Yushchenko Vinnytsya Regional Psychoneurl. Hospital, Neurology Department |
|  | Ukraine | State Institution Ukr. State Research Institute of Medical and Social Problems of Disability, MoH |
|  | Ukraine | O.M. Gorky Donetsk National Medical University, Donetsk Regional Clinical District Unit, Neurology |
|  | Ukraine | Ivano Frankivsk Regional Clinical Hospital, Neurology Department |
|  | Ukraine | Kharkiv Railway Clinical Hospital No 1 of Branch Health Center , Ukrainian Railway , Neurology 3 |
|  | Ukraine | Zaporizhzhya Regional Hospital, Neurology Department |
|  | Ukraine | Poltava Ukrainian Med Stomatological Academy, MV Sklifosovsky Regional Clinical Hospital, Neurology |
|  | Ukraine | Kyiv City Clinical Hospital #4 |
|  | Ukraine | Bukovinian State Medical University, hair of nervous diseases, psychiatry and medical psychology |
|  | Ukraine | Chernihiv Regional Hospital |
|  | Ukraine | State Institution Nat. Research Centre of Radiation Med. of the NAMS of Ukraine |
|  | Ukraine | Regional Clinical Centre of Neurosurgery and Neurology - Department 2 |
|  | Ukraine | S.I. Georgiyevsky Crimean Medical University, Chair of Nervous Diseases; Neurology Department |
|  | Ukraine | MI Odesa Regional Mental Health Medical Centre |
|  | United Kingdom | Salford Royal Hospital - Neurology Department |
|  | United Kingdom | Royal Hallamshire Hospital - Department of Neurology |
|  | USA | University of Colorado - Denver |
|  | USA | Lovelace Scientific Research - MS Department |
|  | USA | MS Center of Greater Washington, P.C. |
|  | USA | Integra Clinical Research - Neurology Department |
|  | USA | Wayne State University - Neurology Department |
|  | USA | Tennessee Neurology Specialists |
|  | USA | Xenoscience, Inc. |
|  | USA | IMMUNOe International Research Center |
|  | USA | Negroski, Sutherland & Hanes Neurology |
|  | USA | The MS Center of Vero Beach |
|  | USA | Oak Clinic for MS - MS Department |
|  | USA | Bhupesh Dihenia, M.D., P.A. |
|  | USA | LSU Health Sciences Center - Neurology Department |
|  | USA | Evergreen Neurological Institute MS Center |
|  | USA | Fullerton Neurological and Headache Center |
|  | USA | Neurological Physicians of Arizona |
|  | USA | Coastal Neurological Medical Group Inc. - Neurology Department |
|  | USA | Collier Neurologic Specialists, LLC |
|  | USA | Virginia Commonwealth University/Medical College of Virginia |
|  | USA | Blue Ridge Research Center LLC |
|  | USA | Meridien Research |
|  | USA | Oklahoma Medical Research Foundation - Rheumatology Department |
|  | USA | Lifetree Clinical Research/Western Neuro Assoc. |
|  | USA | Alpine Clinical Research Center/Associated Neurologists |
|  | USA | University of Miami - Neurology Department |
|  | USA | Neurology Center of San Antonio, P.A. |
|  | USA | Infinity Clinical Research/ MS Research Associates |

| FORTE DB (ClinicalTrials.gov: NCT00337779) | | |
| --- | --- | --- |
| Primary Publications: | Comi G, Cohen JA, Arnold DL, Wynn D, Filippi M, FORTE Study Group. Phase III dose-comparison study of glatiramer acetate for multiple sclerosis. Ann. Neurol. 2011;69:75–82. Available from: http://dx.doi.org/10.1002/ana.22316 | |
| Ethics Statements | The protocol and consent documents were approved by the institutional review boards and ethics committees of the participating centers. Patients provided written informed consent prior to undergoing any study-related procedures.  (Comi et al., 2011). | |
| Site Number | **Country** | **Participating Institution at which Ethics Committees or Institutional Review Boards approved the clinical trial** |
|  | Argentina | Hospital Britanico - Neurology Department |
|  | Argentina | Hospital Central de San Isidro - Neurology Department |
|  | Argentina | Hospital Militar Cordoba - Neurology Office |
|  | Argentina | Instituto de Neurociencias de Rosario - Neurology Office |
|  | Belgium | Academic Hospital University of Leuven - Department of Neurology (MS Department). |
|  | Canada | Foothills Hospital - MS Center |
|  | Canada | Northern Alberta Clinical Trial and Research Centre |
|  | Canada | QE11 Health Science Center - Dalhousie MS Research Unit |
|  | Canada | Montreal Neurological Institute |
|  | Czech Republic | General Faculty Hospital, Department of Neurology |
|  | Czech Republic | Faculty Hospital Ostrava, Department of Neurology |
|  | Czech Republic | Faculty Hospital Hradec Kralove, Department of Neurology |
|  | Czech Republic | Regional Hospital Pardubice, Department of Neurology |
|  | Czech Republic | Faculty Hospital Olomouc Department of Neurology |
|  | Estonia | West Tallinn Central Hospital, Estonian Multiple Sclerosis Centre |
|  | Estonia | University of Tartu, Department of Neurology and Neurosurgery |
|  | Finland | Finn-Medi Research Ltd. |
|  | Finland | Seinajoki Central Hospital - Neurology Department |
|  | Finland | Oulu University Hospital - Neurology Department |
|  | Finland | Hyvinkaa Hospital - Neurology Department |
|  | Finland | Suomen Terveystalo Clinical Research Oy |
|  | France | Hopital Pellegrin - Department de Neurologie - Federation des Neurosciences Cliniques |
|  | France | C.H.U. de Nice - Hopital Pasteur - Service de Neurologie |
|  | France | C.H.U. de la Cote de Nacre - Service de Neurologie Dejerine |
|  | France | Hopital Henri Mondor - Service de Neurologie |
|  | France | C.H.U. de Montpellier - Hopital Gui de Chauliac - Service des Explorations Neurologiques |
|  | France | C.H.U. de Nantes - Hopital Laennec - Centre d'Investigation Clinique - Clinique Neurologique |
|  | Germany | Hans Susemihl-Krankenhaus - Neurologische Abteilung |
|  | Germany | Gemeinschaftspraxis Drs. Franz /Saring/Scheller |
|  | Germany | Judisches Krankenhaus Berlin - Department of Neurology |
|  | Germany | Universitaetsklinikum Eppendorf - Department of Neurology |
|  | Germany | Diakoniekrankenhaus Henriettenstiftung Hannover - Department of Neurology |
|  | Germany | Johannes-Gutenberg-University - Department of Neurology |
|  | Germany | Helios Klinikum Erfurt GmbH - Klinik fuer Neurologie |
|  | Germany | Justus Liebig Universitat Gie en - Department of Neurology |
|  | Germany | Neurologisches Facharztzentrum Berlin |
|  | Germany | Gemeinschaftspraxis Dr. Reifschneider, Unsorg, Dr. Ries |
|  | Germany | Universitaetsklinikum Essen - Neurologische Klinik und Poliklinik |
|  | Germany | Universitaet Ulm - Neurology Department |
|  | Germany | Technische Universitaet Dresden - Neurologische Universitaetsklinik |
|  | Hungary | Jahn Ferenc Hospital, Department of Neurology |
|  | Hungary | Peterfy utcai Hospital, Department of Neurology |
|  | Hungary | Semmelweis University, Department of Neurology |
|  | Hungary | Petz Aladar Teaching Hospital, Department of Neurology |
|  | Hungary | Csolnoky Ferenc Hospital, Department of Neurology |
|  | Hungary | Kaposi Mor Hospital, Department of Neurology |
|  | Israel | Sheba Medical Center-MS Center |
|  | Israel | Barzilai Medical Center-Neurology Department |
|  | Italy | Fondazione Istituto Neurologico Casimiro Mondino - Dipartimento di Clinica Neurologica I |
|  | Italy | Azienda Ospedaliera SAN LUIGI - Centro Regionale Sclerosi Multipla (CRESM) & Neurobiologia Clinica |
|  | Italy | Centro Regionale Sclerosi Multipla c/o Ospedale di Montichiari |
|  | Italy | Fondazione Don Carlo Gnocchi Onlus - IRCCS Centro S. Maria Nascente - U.O.Neurologia - SM |
|  | Italy | Azienda Ospedaliera Universitaria Policlinico Tor Vergata |
|  | Italy | Azienda Ospedaliera Villa Sofia - C.T.O. - U.O. Neurologia |
|  | Italy | Azienda Ospedaliera Universitaria Senese |
|  | Italy | Azienda Ospedaliera S. Camillo-Forlanini - Divisione di Neurologia Centro Sclerosi Multipla |
|  | Italy | Universita degli Studi di Genova - Clinica Neurologica II Dip. Neuroscienze,Oftalmologia e Genetica |
|  | Italy | Universita degli Studi di Ancona Ospedale Regionale Torrette Umberto I - Ist. Malattie Sistema Nerv. |
|  | Italy | Istituto Neurologico Mediterraneo Neuromed (IRCCS) - Dipartimento di Neurologia |
|  | Italy | Fondazione IRCCS Ospedale Maggiore Policlinico,Mangiagalli e Regina Elena -Dip. Scienze Neurologiche |
|  | Italy | A. O. S. Antonio Abate di Gallarate - U.O. Neurologia 2 - Recupero Neurologico - Centro SM |
|  | Italy | Azienda Ospedaliero Universitaria San Giovanni Battista di Torino - Dipartimento di Neuroscienze |
|  | Italy | Fondazione Centro San Raffaele del Monte Tabor - Neuroimaging Research Unit |
|  | Latvia | Maritim Medical Center, Multiple Sclerosis Center |
|  | Lithuania | Vilnius University Hospital Santariski Klinikos, Centre of Neurology |
|  | Lithuania | Hospital of Lithuanian University of Health Sciences Kaunas Clinics |
|  | Netherlands | MS centrum Nijmegen |
|  | Netherlands | Orbis Medical Centre Sittard-Geleen - Department Neurology |
|  | Poland | Centrum Neurologii Klinicznej, Fundacja Przyszlosc Neurologii |
|  | Poland | Wojskowy Instytut Medyczny, Klinika Neurologiczna Centralnego Szpitala Klinicznego MON |
|  | Poland | Samodzielny Publiczny Szpital Kliniczny nr 1, Slaska. Akademia Medyczna, Katedra i Klinika Neuro. |
|  | Poland | Klinika Neurologii, Centralny Szpital Kliniczny MSWiA |
|  | Poland | Klinika Neurologii, Akademicki Szpital Kliniczny im. Jana Mikulicza-Radeckiego we Wroclawiu |
|  | Poland | Pomorskie Centrum Traumatologii, Wojewodzki Szpital Specjalistyczny im. M. Kopernika, Oddz. Neuro. |
|  | Poland | Samodzielny Publiczny Szpital Kliniczny nr 7, SUM GCM im. prof. Leszka Gieca |
|  | Romania | Iasi Clinical Rehabilitation Hospital, Neurology Department |
|  | Romania | University Emergency Hospital Bucharest, Neurology Department |
|  | Romania | Constanta County Clinical Emergency Hospital, Neurology Department |
|  | Romania | Fundeni Clinical Hospital, Neurology Department |
|  | Romania | County Clinical Hospital Cluj, Neurology Department |
|  | Russia | State Budgetary Healthcare Institution City Clinical Hospital #24 of Healthcare Dep. of Moscow |
|  | Russia | State Healthcare Institution Samara M.I.Kalinin Regional Clinical Hospital |
|  | Russia | Pavlov First Saint-Petersburg State Medical University |
|  | Russia | Institution of Russian Academy of Science Human Brain Institute of Russian Academy of Science |
|  | Russia | Saint Petersburg State Healthcare Institution Municipal Multifield Hospital #2 |
|  | Russia | Novosibirsk State Regional Hospital, Center of MS |
|  | Russia | State Healthcare Institution Nizhny Novgorod N.A. Semashko Regional Clinical Hospital |
|  | Russia | Interregional Clinico-Diagnostic Centre |
|  | Russia | Sverdlovskaya Regional Clinical Hospital#1-Neurology Department |
|  | Russia | Perm State Medical Academy - Department of Neurology |
|  | Spain | Hospital De Basurto, Neurology Department |
|  | Spain | Hospital de Cruces, Neurology Department |
|  | Spain | Complejo Hospitalario Carlos Haya, Neurology Department |
|  | Spain | Hospital Universitario de Bellvitge. Servicio de Neurologia |
|  | Spain | Hospital Universitari Vall d'Hebron- Centre d'Esclerosi Multiple de Catalunya (Cemcat) |
|  | Spain | Parc Hospitalari Mari i Julia |
|  | Spain | Hospital Clinic i Provincial de Barcelona - Neurology Department |
|  | Spain | Hospital Universitario La Fe, Neurology Department |
|  | Spain | Hospital Clinico Universitario San Carlos-Neurology Department |
|  | United Kingdom | Royal Hallamshire Hospital - Department of Neurology |
|  | United Kingdom | King's College Hospital - MRC Centre for Neurodegeneration Research, Academic Neuroscience Centre |
|  | United Kingdom | Ninewells Hospital - Department of Neurology |
|  | United Kingdom | The Walton Centre for Neurology and Neurosurgery |
|  | United Kingdom | Charing Cross Hospital - West London Neuroscience Centre |
|  | USA | UC Davis Medical Center - Neurology Department |
|  | USA | Minneapolis Clinic of Neurology - Neurology Department |
|  | USA | Cleveland Clinic Foundation - Mellen Center - Neurology Department |
|  | USA | Clinical and Magnetic Resonance Research Center - MS Department |
|  | USA | MS Clinic of Central Texas - MS Department |
|  | USA | University of Rochester - Neurology |
|  | USA | West County MS Center - Neurology Department |
|  | USA | Ruan M.S. Center - MS Department |
|  | USA | Wake Forest Baptist Health |
|  | USA | Wayne State University - Neurology Department |
|  | USA | Capital Neurology |
|  | USA | University of Kansas Medical Center - Movement Disorders Department |
|  | USA | Indiana University School of Medicine |
|  | USA | Vanderbilt Multiple Sclerosis Center/100 Oaks |
|  | USA | Washington University School of Medicine - Neurology Department |
|  | USA | University of Minnesota - Neurology Department |
|  | USA | Texas Neurology, P.A. - Neurology Department |
|  | USA | Gimbel MS Center - MS Department |
|  | USA | University of Chicago-Department of Neurology |
|  | USA | University of Maryland, School of Medicine - Movement Disorders Department |
|  | USA | MeritCare Neuroscience - MS Department |
|  | USA | Oak Clinic for MS - MS Department |
|  | USA | University of Virginia - Neurology Department |
|  | USA | MS Center at Shepherd - MS Department |
|  | USA | Barrow Neurological Clinic - Neurology Department |
|  | USA | Allied Physicians, Inc. - Ft. Wayne Neurological Center - Neurology Department |
|  | USA | MS Center at Carolinas Medical Center - MS Department |
|  | USA | Thomas Jefferson University-Department of Neurology |
|  | USA | Ohio State University MS Center - MS Department |
|  | USA | Oregon Health Science University - MS Department |
|  | USA | Hospital of the University of Pennsylvania |
|  | USA | Dartmouth Hitchcock Medical Center - MS Center - Neurology Department |

| GA-9003 DB and OL | | |
| --- | --- | --- |
| Primary Publications: | Comi G, Filippi M, Wolinsky JS. European/Canadian multicenter, double-blind, randomized, placebo-controlled study of the effects of glatiramer acetate on magnetic resonance imaging--measured disease activity and burden in patients with relapsing multiple sclerosis. European/Canadian Glatiramer Acetate Study Group. Ann. Neurol. 2001;49:290–7. Available from: http://www.ncbi.nlm.nih.gov/pubmed/11261502 | |
| Ethics Statements | At the pre-enrollment visit eligible patients were informed about all aspects of the study and gave written informed consent. The ethical committees of all participating centers approved the study. (Comi et al., 2001). | |
| Site Number | **Country** | **Participating Institution at which Ethics Committees or Institutional Review Boards approved the clinical trial** |
| 1 | Belgium | Cen. Neurol. Fraiture, Frait/Condoz |
| 2 | Belgium | Clin. Unive. St. Luc Bruss., Brussels |
| 3 | Belgium | Univ. Hosp. Antwerp, Antwerp |
| 4 | Canada | Lon. Healt. Sc. Center, Ontario |
| 5 | Canada | Calgary Gen. Hos., Alberta |
| 6 | Canada | Neur. Inst. Hosp., Montreal, Canada |
| 7 | France | Hop. dela Salpetriere, Paris |
| 8 | France | GIE/IRM CGH Boulloche , Montbeliard |
| 9 | France | Hop. St. Julien, Nancy |
| 10 | Germany | Univ. Heidelberg, Heidelberg |
| 11 | Germany | Knapp. Kranken., Bochum |
| 12 | Germany | Klin. Frd. Schil. Univ., Jena |
| 13 | Italy | Univ. Deg. La Sapienza, Roma |
| 14 | Italy | Univ. di Brescia, Brescia |
| 15 | Italy | Dell. Univ. de Genova, Genova |
| 16 | Italy | Osp. Di Gallarte, Gall-Varese |
| 17 | Italy | Osp. S. Raffaele, Milano |
| 18 | Italy | Inst. Neur. C. Mondino, Pavia |
| 19 | Netherlands | Ignatius Hospital, Breda |
| 20 | United Kingdom | R. Inf. Staffordshire, N. Staffordshire |
| 21 | United Kingdom | Atkin. Morleys Hosp., Wimbledon, London |
| 22 | United Kingdom | Roy. Vict. Infirm., Newcastle-Tyne |
| 23 | United Kingdom | Reg. Neur. Cent. Charing Cross Hosp. London |
| 24 | United Kingdom | Walt. CenNeuro. Neurosur., Liverpool |
| 25 | United Kingdom | Neurosce. Cent. Q. Eliz. Hosp., Birmingham |
| 26 | United Kingdom | Man. Roy. Infirm., Manchester |
| 27 | United Kingdom | Royal Free Hosp., Hampstead |
| 28 | United Kingdom | Royal Infirmary, Leicester |
| 29 | United Kingdom | Q. Med. Cent. Nottingham |

| PreCISe DB and OL (ClinicalTrials.gov: NCT00666224) | | |
| --- | --- | --- |
| Primary Publications: | Comi G, Martinelli V, Rodegher M, Moiola L, Bajenaru O, Carra A, et al. Effect of glatiramer acetate on conversion to clinically definite multiple sclerosis in patients with clinically isolated syndrome (PreCISe study): a randomised, double-blind, placebo-controlled trial. Lancet. Elsevier; 2009;374:1503–11. Available from: http://dx.doi.org/10.1016/S0140-6736(09)61259-9 | |
| Ethics Statements | The protocol and consent documents were approved by the institutional review boards and ethics committees of the participating centres. Patients provided written informed consent before undergoing any study-related procedures (Comi et al., 2009). | |
| Site Number | **Country** | **Participating Institution at which Ethics Committees or Institutional Review Boards approved the clinical trial** |
|  | Argentina | Hospital Britanico & Hospital Central de San Isidro, Neurology Department |
|  | Australia | Queen Elizabeth Hospital- Department of Neurology Clinical Trials Ward 5A |
|  | Australia | Royal Melbourne Hospital-Department of Neurology |
|  | Australia | St. Vincent Hospital |
|  | Austria | Universitatsklinikum St. Poelten - Neurologie |
|  | Austria | Medizinische Universitat Graz, Universitatsklinik fur Neurologie |
|  | Denmark | Copenhagen University Hospital, Rigshospitalet 2082 |
|  | Denmark | Glostrup Amtssygehus, Neurological Department |
|  | Finland | University of Tampere, Medical School-Finn Medi 3 |
|  | Finland | Suomen Terveystalo Clinical Reserch Oy |
|  | Finland | Oulu University Hospital - Neurology Department |
|  | France | C.H.U. de Toulouse - Hopital Purpan - Service de Neurologie |
|  | France | Hopital Neurologique - Groupe Hospitalier Est - Service des Essais Therapeutiques du Pr. Confavreux |
|  | France | Hopital La Pitie Salpetriere - Federation de Neurologie |
|  | France | Hopital Pontchaillou - Clinique Neurologique |
|  | France | Hopital Pellegrin - Departement de Neurologie - Federation des Neurosciences Cliniques |
|  | France | C.H.U. de Clermont-Ferrand - Hopital Gabriel Montpied - Service de Neurologie |
|  | France | C.H.U. La Timone - Service de Neurologie |
|  | France | C.H.U. de Nice - Hopital Pasteur - Service de Neurologie |
|  | France | Hopital Jean Minjoz - Service de Neurologie |
|  | Germany | Diakoniekrankenhaus Henriettenstiftung Hannover - Department of Neurology |
|  | Germany | Hans Susemihl-Krankenhaus - Neurologische Abteilung |
|  | Germany | Helios Klinikum Erfurt GmbH - Klinik fuer Neurologie |
|  | Germany | Johannes-Gutenberg-University, Judisches Krankenhaus Berlin - Department of Neurology |
|  | Germany | Julius-Maximilians-Universitat - Neurologische Klinik |
|  | Germany | Justus Liebig Universitat Giessen - Department of Neurology |
|  | Germany | Klinikum der Otto-von-Guericke Universitat - Neurologische Universitatsklinik |
|  | Germany | Universitaet Ulm - Neurology Department |
|  | Germany | Universitaetsklinikum Duesseldorf, Eppendorf, Koln, Muenster |
|  | Hungary | Jahn Ferenc Hospital, Department of Neurology |
|  | Hungary | Josa Andras County Hospital, Department of Neurology |
|  | Hungary | Kaposi Mor Hospital, Department of Neurology |
|  | Hungary | Kenezy Hospital and Polyclinic, Department of Neurology |
|  | Hungary | Semmelweis University, Department of Neurology |
|  | Hungary | Szent Imre Hospital, Department of Neurology |
|  | Hungary | Uzsoki utcai Hospital, Department of Neurology |
|  | Italy | Azienda USL di Parma - Ospedale di Fidenza - San Secondo Pse - U.O. Neurologia |
|  | Italy | Universita degli Studi di Genova - Clinica Neurologica II Dip. Neuroscienze,Oftalmologia e Genetica |
|  | Italy | A. O. S. Antonio Abate di Gallarate - U.O. Neurologia 2 - Recupero Neurologico - Centro SM |
|  | Italy | Fondazione Istituto Neurologico Casimiro Mondino - Dipartimento di Clinica Neurologica I |
|  | Italy | Fondazione Centro S. Raffaele del Monte Tabor - Dipartimento Neurologico |
|  | Italy | Universita degli Studi di Napoli Federico II - Dipartimento Scienze Neurologiche |
|  | Italy | Fondazione Don Carlo Gnocchi Onlus - IRCCS Centro S. Maria Nascente - U.O. Neurologia - SM |
|  | Italy | Azienda Ospedaliera S. Camillo-Forlanini - Divisione di Neurologia Centro Sclerosi Multipla |
|  | Italy | Universita degli Studi di Catania - Azienda Policlinico - Clinica Neurologica I - Centro SM |
|  | Italy | Istituto Neurologico Mediterraneo Neuromed (IRCCS) - Dipartimento di Neurologia |
|  | Italy | Universita degli Studi di Ancona Ospedale Regionale Torrette Umberto I - Ist. Malattie Sistema Nerv. |
|  | Italy | Azienda Ospedaliera SAN LUIGI - Centro Regionale Sclerosi Multipla (CRESM) & Neurobiologia Clinica |
|  | New Zealand | Auckland City Hospital |
|  | Norway | Haukeland University Hospital |
|  | Romania | Clinical Rehabilitation Hospital Cluj, Neurology Department |
|  | Romania | County Clinical Hospital Timisoara, Targu Mures, Neurology Department |
|  | Romania | University Emergency Hospital Bucharest, Neurology Department |
|  | Romania | ELIAS Emergency Clinical Hospital, Neurology Department |
|  | Spain | Complejo Hospitalario Carlos Haya-Neurology Department |
|  | Spain | Hospital de Cruces & Hospital De Basurto, Neurology Department |
|  | Spain | Hospital Virgen Macarena |
|  | Spain | Hospital Universitario de Bellvitge. Servicio de Neurologia |
|  | Spain | Hospital Universitari Vall d'Hebron- Centre d'Esclerosi Multiple de Catalunya (Cemcat) |
|  | Spain | Clinica Puerta de Hierro |
|  | Spain | Parc Hospitalari Mari i Julia |
|  | Sweden | MS Centrum Forskningsenhet-Hus T |
|  | Sweden | Huddinge University Hospital-Department of Neurology |
|  | United Kingdom | Royal Victoria Infirmary - Neurology Department |
|  | United Kingdom | Queen's Medical Centre, Division of Clinical Neurology |
|  | United Kingdom | University Hospital of North Staffordshire NHS Trust |
|  | United Kingdom | Royal Hallamshire Hospital - Department of Neurology |
|  | United Kingdom | King's College Hospital - MRC Centre for Neurodegeneration Research, Academic Neuroscience Centre |
|  | United Kingdom | Leicester Royal Infirmary - Neurology Department |
|  | United Kingdom | The Walton Centre for Neurology and Neurosurgery |
|  | USA | Geisinger Medical Center - Neurology Department |
|  | USA | Multiple Sclerosis Center of Atlanta |
|  | USA | Henry Ford Hospital - Neurology Department |
|  | USA | University of New Mexico Health Sciences Ctr. |
|  | USA | State University of NY at Stony Brook - Neurology Department |
|  | USA | Wayne State University - Neurology Department |
|  | USA | Ohio State University MS Center - MS Department |
|  | USA | Michigan Institute for Neurological Disorders - Neurology Department |
|  | USA | University of Rochester - Neurology |
|  | USA | Maryland Center for MS - MS Department |

| BRAVO DB (ClinicalTrials.gov: NCT00605215) | | |
| --- | --- | --- |
| Primary Publications: | Vollmer TL, Sorensen PS, Selmaj K, Zipp F, Havrdova E, Cohen JA, et al. A randomized placebo-controlled phase III trial of oral laquinimod for multiple sclerosis. J. Neurol. 2014; 261:773–83. Available from: http://dx.doi.org/10.1007/s00415-014-7264-4 | |
| Ethics Statements | The BRAVO protocol was approved by local Ethics Committees/Institutional Review Boards (EC/IRB), including Committees on Human Experimentation. Patients provided written informed consent before participating in study procedures. A Data Monitoring Committee periodically reviewed data to ensure patient welfare. (Vollmer et al. 2014) | |
| Site Number | **Country** | **Participating Institution at which Ethics Committees or Institutional Review Boards approved the clinical trial** |
|  | Bulgaria | 1st MHAT, Sofia |
|  | Bulgaria | CCB Medical Institute - Ministry of Interiority |
|  | Bulgaria | MHAT Dr. Stefan Cherkezov , Department of Neurology |
|  | Bulgaria | MHAT Prof. Stoyan Kirkovich AD |
|  | Bulgaria | MHAT Sveti Georgi |
|  | Bulgaria | MHAT NCH EAD |
|  | Bulgaria | MHAT Ruse, 1st Department of Neurology |
|  | Bulgaria | MHAT Shumen, Neurology Department |
|  | Bulgaria | MHAT Tokuda Hospital Sofia AD, Department of Neurology |
|  | Bulgaria | MMA HAT Sofia - Clinic of Functional Diagnostics of Nervous System |
|  | Bulgaria | MMA HAT Sofia - Clinic of Neurology |
|  | Bulgaria | Multiprofile Hospital for Active Treatment Sveta Marina EAD |
|  | Bulgaria | SHATNPsy - St. Naum EAD |
|  | Bulgaria | SHATNPsy Sveti Naum EAD |
|  | Bulgaria | UMHAT Dr. Georgi Stranski |
|  | Bulgaria | UMHAT St. Ivan Rilski EAD |
|  | Bulgaria | UMHAT Pleven - First Clinic of Neurology |
|  | Croatia | Clinical Hospital Center Zagreb |
|  | Croatia | Clinical Hospital Sestre milosrdnice |
|  | Croatia | Clinical Hospital Osijek |
|  | Croatia | General Hospital Varazdin |
|  | Croatia | Clinical Hospital Sveti Duh |
|  | Czech Republic | General Faculty Hospital, Department of Neurology |
|  | Czech Republic | Faculty Hospital Olomouc Department of Neurology |
|  | Czech Republic | Faculty Hospital Motol, Department of Neurology |
|  | Czech Republic | Hospital Teplice, Neurology department |
|  | Czech Republic | Neurology Department St. Anne`s University Hospital Brno |
|  | Estonia | West Tallinn Central Hospital, Estonian Multiple Sclerosis Centre |
|  | Estonia | East Viru Central Hospital |
|  | Estonia | University of Tartu, Department of Neurology and Neurosurgery |
|  | Georgia | LTD Petre Sarajishvili Institute of Neurology |
|  | Georgia | LTD Medical Center CITO |
|  | Georgia | Research Institute of Clinical Medicine |
|  | Germany | Universitaet Ulm - Neurology Department |
|  | Germany | Diakoniekrankenhaus Henriettenstiftung Hannover - Department of Neurology |
|  | Germany | Charite Campus Benjamin Franklin Klinik |
|  | Germany | Charite Campus Benjamin Franklin, Hochschulambulanz fuer Neurologie |
|  | Germany | Krankenhaus Hohe Warte Bayreuth gGmbH |
|  | Germany | Charite, Universitaetsmedizin Berlin |
|  | Israel | Sheba Medical Center, MS Center |
|  | Israel | Hadassah Medical Center, Neurology Department |
|  | Italy | Fondazione PTV, Policlinico Tor Vergata |
|  | Italy | Universita Cattolica del Sacro Cuore - Policlinico A. Gemelli - Dip. Neuroscienze - UOC Neurologia |
|  | Italy | Azienda Ospedaliera S. Camillo-Forlanini - Divisione di Neurologia Centro Sclerosi Multipla |
|  | Italy | Fondazione Istituto San Raffaele - G. Giglio di Cefalu - Unita Operativa Neurologia |
|  | Italy | Ospedale Clinicizzato - SS. Annunziata |
|  | Italy | Universita degli Studi di Napoli Federico II - Dipartimento Scienze Neurologiche |
|  | Italy | Ospedale Bellaria - Azienda USL di Bologna |
|  | Italy | Azienda Ospedaliera di Rilievo Nazionale e di Alta Specializzazione Garibaldi |
|  | Lithuania | Republican Siauliai Hospital |
|  | Lithuania | Hospital of Lithuanian University of Health Sciences Kaunas Clinics |
|  | Macedonia | Neurology Clinic, Clinical Centre |
|  | Macedonia | Department of preclinical and clinical pharmacology and toxicology, Medical Faculty |
|  | Macedonia | Municipal Clinical Hospital |
|  | Poland | Prywatny Gabinet Neurologiczny Waldemar Brola |
|  | Poland | SPZOZ Uniwersytecki Szpital Kliniczny Nr 1 im. Norberta Barlickiego UM w Lodzi |
|  | Poland | COPERNICUS Podmiot Leczniczy Sp. z o.o. |
|  | Poland | SPZOZ Wojewodzki Szpital Specjalistyczny w Olsztynie |
|  | Poland | Centrum Medyczne EUROMEDIS |
|  | Poland | SP ZOZ Szpital Specjalistyczny w Koscierzynie, Oddzial Neurologiczny |
|  | Poland | Samodzielny Publiczny Specjalistyczny Szpital Zachodni im. Jana Pawla II |
|  | Poland | Niepubliczny Zaklad Opieki Zdrowotnej NOVO-MED |
|  | Poland | Akademicki Szpital Kliniczny im. Jana Mikulicza-Radeckiego we Wroclawiu |
|  | Poland | Klinika Neurologii, Jana Mikulicza-Radeckiego we Wroclawiu |
|  | Poland | Instytut Psychiatrii i Neurologii, II Klinika Neurologiczna |
|  | Poland | Niepubliczny Zaklad Opieki Zdrowotnej KENDRON |
|  | Poland | Uniwersyteckie Centrum Kliniczne, Klinika Neurologii Doroslych |
|  | Poland | NZOZ Centrum Medyczne DENDRYT |
|  | Poland | Klinika Neurologiczna Centralnego Szpitala Klinicznego MON |
|  | Poland | Wojskowy Instytut Medyczny, Centralnego Szpitala Klinicznego MON |
|  | Poland | SP Centralny Szpital Kliniczny |
|  | Poland | Szpital Specjalistyczny im. L. Rydygiera w Krakowie |
|  | Poland | NZOZ Przychodnia Neurologiczna Belon, Krynicka, Sobkowiak-Osinska s.c. |
|  | Poland | RESMEDICA Elzbieta Jasinska |
|  | Romania | University Emergency Hospital Bucharest, Neurology Department |
|  | Romania | Fundeni Clinical Hospital, Neurology Department |
|  | Romania | County Clinical Hospital Cluj, Neurology Department |
|  | Romania | Iasi Clinical Rehabilitation Hospital, Neurology Department |
|  | Romania | Constanta County Clinical Emergency Hospital, Neurology Department |
|  | Romania | Central Military Clinical Emergency Hospital Dr. Carol Davila , Neurology Department |
|  | Romania | County Clinical Emergency Hospital Sibiu |
|  | Romania | Prof. Dr. Agrippa Ionescu Clinical Emergency Hospital |
|  | Romania | Neuropsychiatry Clinical Hospital Craiova, Neurology Department |
|  | Russia | State Budgetary Healthcare Institution, Moscow |
|  | Russia | City Clinical Hospital #24, Healthcare Dep. of Moscow |
|  | Russia | RSMU, Department of Neurology |
|  | Russia | Institution of Russian Academy of Science |
|  | Russia | Human Brain Institute of Russian Academy of Science |
|  | Russia | Pavlov First Saint-Petersburg State Medical University |
|  | Russia | Leningrad Regional Hospital - Neurology Department |
|  | Russia | State Healthcare Institution Samara M.I.Kalinin Regional Clinical Hospital |
|  | Russia | Novosibirsk State Regional Hospital, Center of MS |
|  | Russia | Institution of Russian Academy of Medical Sciences Scientific Centre of Neurology of RAMS (SCN RAMS) |
|  | Russia | SRI of Clinical and Experimental Lymphology, Siberian Branch of RAMS, Therapy Department |
|  | Russia | State Healthcare Institution Territorial Clinical Hospital |
|  | Russia | Bashkir State Medical University of Roszdrav |
|  | Slovakia | Faculty Hospital Bratislava, Department of Neurology |
|  | Slovakia | Faculty Hospital Bratislava Ruzinov, Department of Neurology |
|  | Slovakia | Faculty Hospital Nitra, Department of Neurology |
|  | Slovakia | Hospital in Zilina, Department Of Neurology |
|  | South Africa | University of Cape Town, Division of Neurology |
|  | South Africa | Groote Schuur Hospital |
|  | South Africa | Charlotte Maxeke Johannesburg Academic Hospital - Division of Neurology |
|  | South Africa | Willows Medical Centre, Neurology |
|  | South Africa | Rosebank Medical and Dental Centre, Neurology |
|  | Spain | Hospital Universitari Vall d'Hebron- Centre d'Esclerosi Multiple de Catalunya (Cemcat) |
|  | Spain | Hospital Virgen Macarena |
|  | Spain | Hospital Universitario de Bellvitge. Servicio de Neurologia |
|  | Spain | Complejo Hospitalario Carlos Haya-Neurology Department |
|  | Spain | Hospital Universitario 12 de Octubre-Neurology Department |
|  | Spain | Hospital de Tortosa Verge de la Cinta-Neurology Department |
|  | Spain | Hospital de Figueres-Fundacio Salut Emporda-Neurology Department |
|  | Ukraine | Lviv National Medical University, Chair of Neurology |
|  | Ukraine | Lviv Regional Clinical Hospital, Neurology Dep |
|  | Ukraine | Yushchenko Vinnytsya Regional Psychoneurl. Hospital, Neurology Department |
|  | Ukraine | State Institution Ukr. State Research Institute of Medical and Social Problems of Disability, MoH |
|  | Ukraine | O.M. Gorky Donetsk National Medical University, Donetsk Regional Clinical District Unit, Neurology |
|  | Ukraine | Ivano Frankivsk Regional Clinical Hospital, Neurology Department |
|  | Ukraine | Kharkiv Railway Clinical Hospital No 1 of Branch Health Center , Ukrainian Railway , Neurology 1 |
|  | Ukraine | Zaporizhzhya Regional Hospital, Neurology Department |
|  | Ukraine | Kharkiv Railway Clinical Hospital No 1 of Branch Health Center , Ukrainian Railway , Neurology 3 |
|  | Ukraine | Institute of Neurology Psychiatry and Neurology of AMS Ukraine Department of Neuroinfections and MS |
|  | Ukraine | Municipal Institution City Clinical Hospital #6 |
|  | Ukraine | Odessa Regional Clinical Hospital, Department of Neurosurgery |
|  | Ukraine | Poltava Ukrainian Med Stomatological Academy, MV Sklifosovsky Regional Clinical Hospital, Neurology |
|  | Ukraine | Kyiv City Clinical Hospital #4 |
|  | Ukraine | Bukovinian State Medical University |
|  | Ukraine | Chernihiv Regional Hospital |
|  | USA | Barrow Neurological Clinic - Neurology Department |
|  | USA | University of California, Davis |
|  | USA | Ayres and Associates Clinical Trials, Upper Valley Neurology |
|  | USA | Caribbean Center for Clinical Research |
|  | USA | Providence MS Center |
|  | USA | Cleveland Clinic Foundation - Mellen Center - Neurology Department |
|  | USA | Neurological Research Institute |
|  | USA | Raleigh Neurology Associates - Neurology Department |
|  | USA | OSFMG - Neurology |
|  | USA | MultiCare Health System (previously Neurology & Neurosurgery Associates of Tacoma Inc., P.S.) |
|  | USA | Neurological Services of Orlando (formerly MS Care Center of NSO) |
|  | USA | Indiana University School of Medicine |
|  | USA | Neurology and Neuroscience Associates |
|  | USA | LSU Health Sciences Center - Neurology Department |
|  | USA | Dent Neurological Group LLP - Neurology Department |
|  | USA | Alabama Neurology Associates |
|  | USA | Maryland Center for MS - MS Department |
|  | USA | Blue Ridge Research Center LLC |
|  | USA | Neuro Therapeutics, Inc. |
|  | USA | Upstate Neurology Consultants, LLC |
|  | USA | MS Center at Shepherd - MS Department |
|  | USA | Territory Neurology & Research Institute |
|  | USA | LSUHSC Epilepsy Center of Excellence |
|  | USA | North Valley Neurology & Sleep/Hope Research Institute |
|  | USA | University of Colorado - Denver |
|  | USA | St Thomas Health - St Thomas Neurology Specialists |
|  | USA | Lovelace Scientific Research - MS Department |
